# Upgrade of Weak σ‐Hole Bond Donors via Cr(CO)_3_ Complexation

**DOI:** 10.1002/chem.202404570

**Published:** 2025-01-28

**Authors:** Arun Dhaka, Roberta Beccaria, Andrea Pizzi, Elena Yu. Tupikina, Vadim Yu. Kukushkin, Giuseppe Resnati

**Affiliations:** ^1^ NFMLab, Dept. Chemistry, Materials, and Chemical Engineering “Giulio Natta” Politecnico di Milano via L. Mancinelli 7 I-20131 Milano Italy; ^2^ Institute of Chemistry Saint Petersburg State University Universitetskaya Emb 7/9 199034 Saint Petersburg Russian Federation

**Keywords:** Halogen bond, σ-hole, Complexation, Activation, Chlorine

## Abstract

Molecular recognition mediated by σ‐hole interactions is enhanced as the electrostatic potential at the σ‐hole becomes increasingly positive. Traditional methods to strengthen σ‐hole donor ability of atoms such as halogens often involve covalent modifications, such as, introducing electron‐withdrawing substituents (neutral or positively charged) or electrochemical oxidation. Metal coordination, a relatively underexplored approach, offers a promising alternative. In this study, η^6^‐coordination of Cr(CO)_3_ to haloarenes, a neutral system, is demonstrated to significantly increase the electrophilic character of halogen bond donors, enabling weak donors such as chloroanisole to form short and directional Cl⋅⋅⋅O halogen bonds. Structural characterization using single‐crystal X‐ray diffraction and computational analysis of a series of η^6^‐Cr(CO)_3_‐coordinated haloarenes provides evidence for this enhancement. Furthermore, the effect is shown to extend to other heteroatomic substituents on the coordinated arene, e. g., other halogen atoms as well as elements of groups 16, 15, and 14 of the periodic table, broadening the scope of this approach.

## Graphical abstract

Crystallographic studies and theoretical calculation prove that the η^6^‐coordination of the neutral Cr(CO)_3_ moiety by arenes is a general approach for activating the σ‐hole donor ability of heteroatoms appended to the arene.

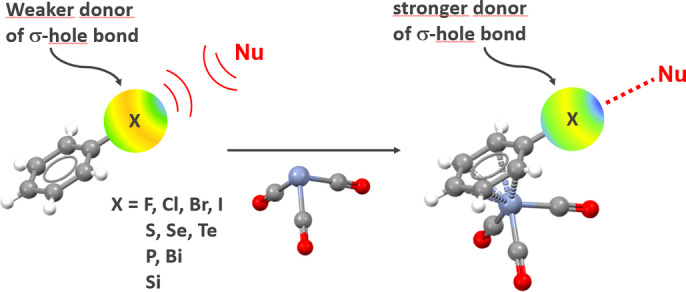

## Introduction

σ‐Hole bonds are the attractive interactions between donors of electron density (e. g., nucleophiles such as O and N atoms) and the region(s) of depleted electron density (σ‐holes, electrophilic sites) that atoms present opposite to the σ covalent bonds they are involved in.^[1]^

Typically, the strength of these interactions increases when the electron withdrawing ability of the substituents bonded to the atom bearing the σ‐hole is increased. Most frequently, a covalent approach is employed to enhance σ‐hole bonds strength on a given atom. For instance, by inserting electron withdrawing substituents (e. g., fluorine atoms or nitro or cyano groups) close to the atom bearing the σ‐hole, the electrostatic potential at the σ‐hole becomes more positive and the resulting σ‐hole bonds stronger.[^2^, ^3^, ^4^, ^5^] Similarly, in compounds wherein the atom bearing the σ‐hole is appended to, or part of, an N‐/S‐heterocyclic system, the σ‐hole bonds strength increases when the heterocyclic system is protonated,[^6^, ^7^] alkylated,[^8^, ^9^, ^10^, ^11^, ^12^, ^13^] or electrochemically oxidized.^[14]^ Less frequently, a noncovalent (supramolecular) approach has been employed,^[15]^ e. g., the ability of haloarenes to form halogen bonds (HaBs), (the σ‐hole interactions wherein the electrophile is a halogen)^[16]^ has been boosted by forming π‐π stacking complexes with electron deficient arenes.^[17]^ In other cases, coordination of derivatives of metal salts was used, e. g., HaB and chalcogen bond (ChB)^[18]^ formation are promoted by binding Pd(II) chloride to the nitrogen of halopyridines^[8]^ or directly to the chalcogen atom;[^19^, ^20^] also, the ability of haloarenes to form HaB is promoted by η^6^‐coordination of *cationic metal moieties* (i. e., cyclopentadienyl‐Ru(II)) to the arene.^[21]^ We decided to assess how strong the electron density depletion at the σ‐hole(s) of heteroatoms appended to an arene is when *neutral metal moieties* are η^6^‐coordinated to the arene. Specifically, we investigated if the depletion of electron density at an appended heteroatom is strong enough to make the resulting σ‐hole bonds persistent supramolecular interactions even when in the uncoordinated system the heteroatom is a weak σ‐hole bond donor.

**Scheme 1:**
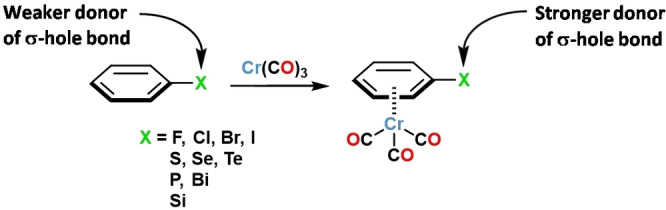
Cr(CO)_3_ complexation by substituted arenes boosts the ability of substituents to form σ‐hole bonds.

Here we report experimental and computational results showing that the HaB donor ability of chlorine in η^6^‐chloroarene‐Cr(CO)_3_ derivatives is boosted to the point that short[^22^, ^23^] Cl⋅⋅⋅O HaBs close to linearity are formed by HaB donors as weak as 4‐chloroanisole. Computation indicates that Cr(CO)_3_ complexation by heteroatom substituted benzene derivatives increases the ability to form σ‐hole bonds also in heteroatoms other than chlorine (e. g., F, Br, I, and elements of groups 16, 15, and 14 of the periodic table, Scheme 1).

## Results and Discussion

**Figure 1:**
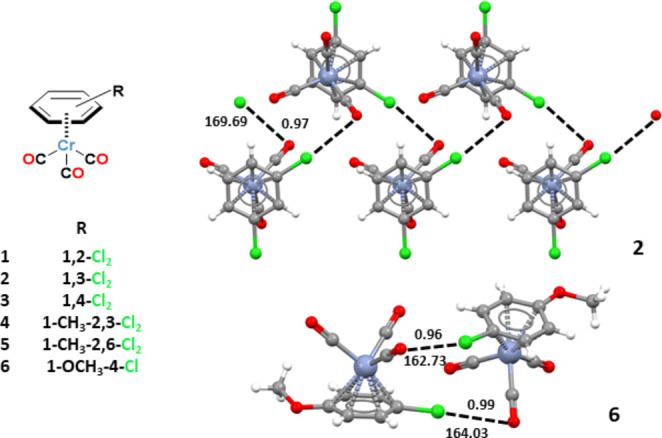
Structural formulas of complexes **1**–**6** (left). Supramolecular architectures assembled by Cl⋅⋅⋅O contacts (black dashed lines) in complexes **2** (infinite chain; top, right) and **6** (dimer; bottom, right); Nc values and angles (°) of the Cl⋅⋅⋅O contacts are given; colour coding: grey, carbon; whitish, hydrogen; red, oxygen; green, chlorine; sky blue, chromium.

*Ortho*‐dichlorobenzene‐Cr(CO)_3_ (**1**), its *meta*‐ and *para*‐ analogues **2** and **3**, as well as 2,3‐ and 2,6‐dichloro‐toluene Cr(CO)_3_ **4** and **5**, and *p*‐chloroanisole‐Cr(CO)_3_ **6** (Figure 1) were prepared by refluxing the respective chloroarene and Cr(CO)_6_ in dioxane/diglyme or (*n*‐Bu)_2_O/THF mixtures.[^24^, ^25^, ^26^] Crystals suitable for single crystal X‐ray analyses were obtained by slow isothermal evaporation of *n*‐hexane solutions (Tables S2–S7). Consistent with the non‐minor acidity of hydrogen atoms in arene‐Cr(CO)_3_ complexes,[^27^, ^28^] a network of H⋅⋅⋅O and H⋅⋅⋅Cl hydrogen bonds (HBs) characterizes the packing of these crystals (Figures S3‐S5). Despite the possible structural constrains resulting from these networks, short intermolecular Cl⋅⋅⋅O contacts are present in all six structures revealing that the Cl atoms electrophilicity is strong enough to make the Cl⋅⋅⋅O supramolecular synthon a persistent feature of the interactional landscape of chloroarene Cr(CO)_3_ complexes in the solid.

Analyses of the Cambridge Structural Database (CSD) unequivocally indicates that chloroarenes are weak HaB donors, only 5.3 % of the structures which can form a short Cl⋅⋅⋅O contact shows the presence of interactions which can be considered Cl⋅⋅⋅O HaBs (Cl⋅⋅⋅O distance≤335 pm, C−Cl⋅⋅⋅O angle≥160°). For the three isomeric dichlorobenzenes the percentage considered above lowers to 4.1 % (Table S1) and the angular distribution of the contacts (Figure S2) reveals that the HaB contribution to the formation of the Cl⋅⋅⋅O contacts is quite small, if any. Of note, the three isomeric dichlorobenzes **1**–**3** show the presence of one or more short Cl⋅⋅⋅O contacts fulfilling the geometric and electronic requirements for being considered HaBs. The corresponding Cl⋅⋅⋅O separations can be as short as 318.7 pm (the C1A−Cl1A⋅⋅⋅O2B interaction in the *para* isomer **3**, the corresponding normalized contact (Nc)^[29]^ is 0.95) and the C−Cl⋅⋅⋅O angles have a tendency to be close to linearity (the values for the most linear contacts in **1**, **2**, and **3** are 173.49°, 169.69°, and 161.02° respectively). These short contacts form supramolecular architectures with different topologies, e. g., dimers in the *ortho* isomer **1** (Figure S3) and infinite chains in the *meta* isomer **2** (Figure 1; top right).

In crystals of pure *o*‐, *m*‐, and *p*‐dichlorobenzene isomers, the region of excess electron density on a chlorine atom (i. e., the belt orthogonal to the C−Cl covalent bond formed by the three lone pairs) might form an attractive interaction with the region of depleted electron density on another chlorine atom (i. e., the cap opposite to the C−Cl covalent bond) and C−Cl⋅⋅⋅Cl HaBs might be produced.^[30]^ *p*‐Cl_2_‐C_4_H_4_ crystallizes in three polymorphs[^31^, ^32^] and none of them shows the presence of a C−Cl⋅⋅⋅Cl HaBs even at distances 20 pm longer than two times the Cl van der Waals radius. Via in situ cryocrystallization^[33]^ and high pressure^[34]^ *m*‐ and *o*‐Cl_2_‐C_4_H_4_O afford the same polymorphs which present quite long C−Cl⋅⋅⋅Cl HaBs (corresponding Nc values are 1.03 and 0.99). These C−Cl⋅⋅⋅Cl contacts, compared with C−Cl⋅⋅⋅O ones in complexes **1**–**3**, supports an increased HaB donor ability of the halogen atoms on Cr(CO)_3_ coordination (see onwards).

These results encouraged us to assess the ability of Cr(CO)_3_ complexation to activate short and linear Cl⋅⋅⋅O interactions formation by chloroarene‐Cr(CO)_3_ complexes which would serve as more demanding probes than dichloroarenes **1–3**. We considered the complexes **4**–**6** wherein the presence of electron donor substituents on the benzene ring increases the electron density on the Cl atoms, namely makes them weaker HaB donors (see onward). Interestingly, also in these derivatives Cl⋅⋅⋅O contacts are present and, similar to **1**–**3** complexes, they complement the H⋅⋅⋅O and H⋅⋅⋅Cl HBs in determining the adopted crystal packing. Some of these contacts are quite short and C−Cl⋅⋅⋅O angles are close to linearity (hereinafter these interactions are addressed as linear contacts). For instance, in the dichlorotoluene derivative **5** the Cl1⋅⋅⋅O3 interaction is 307.7 pm long (Nc=0.92) and in the isomeric complex **4** the C3‐Cl2 A⋅⋅⋅O2 angle is 170.71° (Figures S4, S5). Remarkably, even the chlorine atom of the quite electron rich chloroanisole complex **6** is activated enough to form a Cl⋅⋅⋅O contact with an Nc value of 0.96 (321.4 pm) (Figure 1; bottom right).

**Figure 2:**
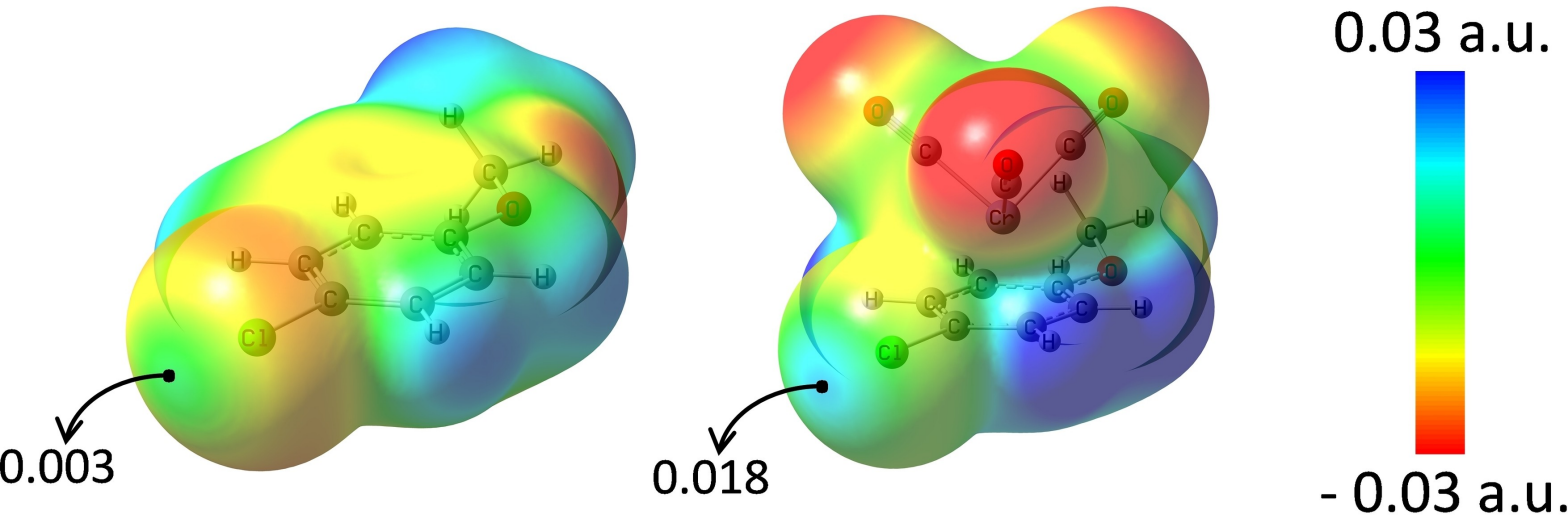
MEP surface (isovalue of electron density 0.001 a.u.) for 4‐Cl‐anisole (left) and its Cr(CO)_3_ complex **6** (right). V_S,max_ at Cl (a.u.) are reported; geometries were optimized in the gas phase with relativistic (spin‐orbit) corrections (PW6B95‐D3‐DKH/def2‐QZVPPD).

Calculation of the molecular electrostatic potentials (MEPs) for Cr(CO)_3_ coordination products **1**–**6** and for the corresponding pure chlorobenzene compounds evidences the role of the Cr(CO)_3_ coordination in activating the ability of the chlorine atoms to act as dependable HaB donors. The depletion of the electron density on the whole chloroarene molecules caused by the η^6^‐coordination of the neutral Cr(CO)_3_ moiety deepened the σ‐hole on Cl atoms. The electrostatic potential at the Cl σ‐holes is approximately 0.003 a.u. for chloroanisole (Figure 2) and 0.009‐0.012 a.u. for the other five uncomplexed chloroarenes (Figures S6, S7). On η^6^‐coordination of Cr(CO)_3_, the potential becomes six and two times more positive for the chloroarene‐Cr(CO)_3_ complexes **6** and **1**–**5**, respectively. This suggests a significant increase of the HaB donor ability of chlorine and accounts for the recurring presence of HaBs in **1**–**6** crystals.

**Figure 3:**
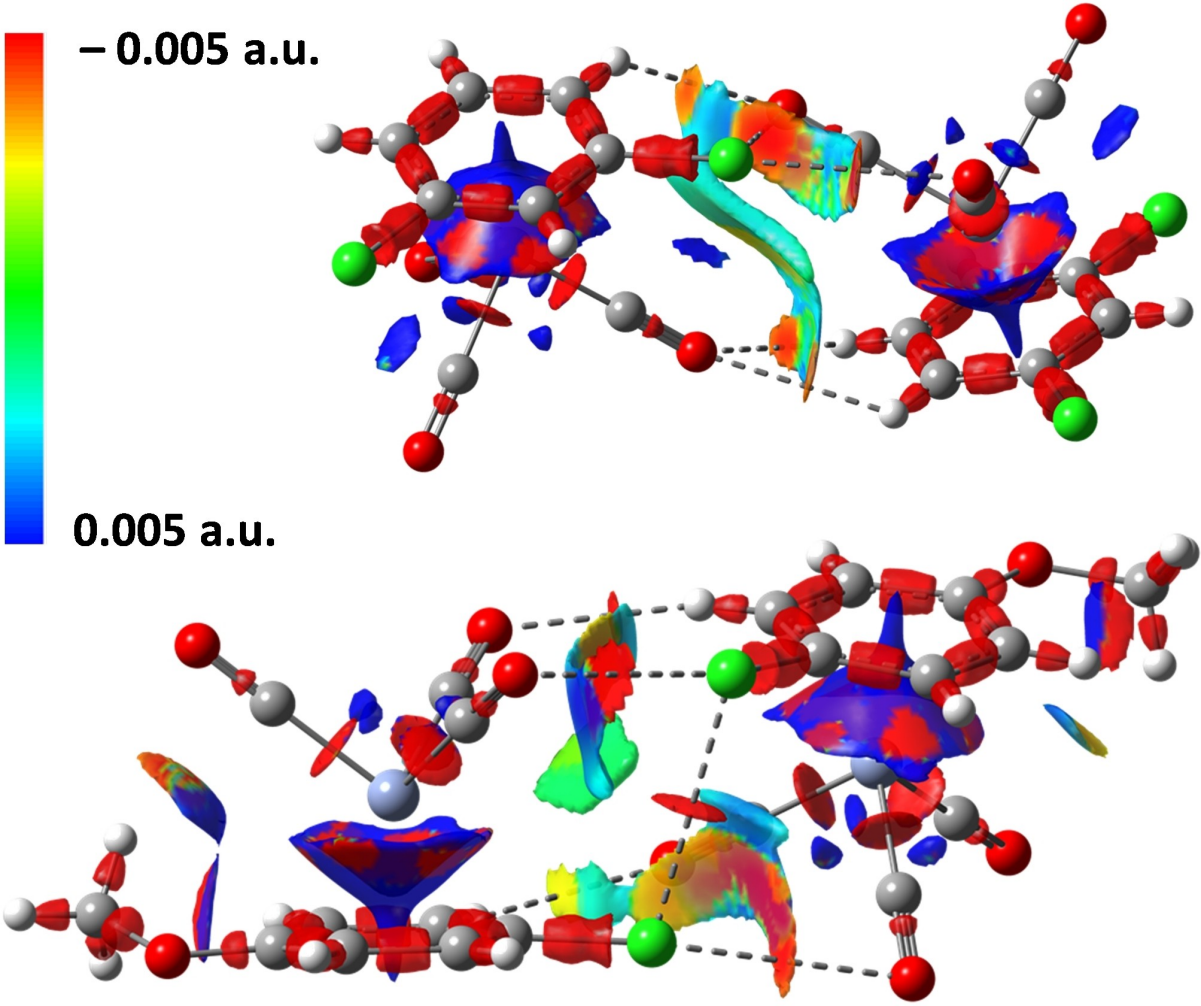
IRI isosurfaces (1.0, mapped by sign(λ_2_(ρ) for dimers of chloroarene‐Cr(CO)_3_ complexes **2** (top) and **6** (bottom). The geometries from the crystals (without optimization) were used.

The Interaction Region Indicator (IRI) and the Quantum Theory of Atoms In Molecules (QTAIM) prove the attractive nature of the linear Cl⋅⋅⋅O contacts observed in the crystals. The IRI isosurfaces were calculated for dimers of chloroarene‐Cr(CO)_3_ complexes **1**–**6** formed *via* the short and linear C−Cl⋅⋅⋅O interactions observed in the crystals (Figure 3 and S8). These analyses gave information on the overall interactional landscape of the complexes, e. g., peculiarities of sign(λ_2_)ρ distribution in the region between Cr(CO)_3_ and benzene ring (red and blue colours on cone surfaces) evidence a major electron density redistribution upon Cr(CO)_3_ coordination. Red surfaces are invariably present along the short and linear Cl⋅⋅⋅O interactions proving their stabilizing nature. Red surfaces are typically found also along other short contacts (e. g., the HBs mentioned above) confirming the synergistic action of various and different interactions in determining the adopted crystal packing. QTAIM analysis identify the expected (3; −1) bond critical points (CPs) for the short and linear Cl⋅⋅⋅O contacts and the electron density parameters at these points (∇^2^ρ sign, ρ values, proximity to zero of H values, Table S8) are typical for moderate HaBs. Interestingly, the calculated electron density at the bond critical point is, in most cases, larger for Cl⋅⋅⋅O contact than that observed for other interaction (e. g., H⋅⋅⋅O HBs).

Having definitively proven that η^6^‐coordination of the neutral Cr(CO)_3_ moiety substantially increases the HaB donor ability of chloroarenes so that they become dependable HaB donors, we decided to extend our analyses and establish if this coordination performs similarly with other weak donors of σ‐hole interactions appended to the arene.

The structures of [(1‐chloro‐2‐(1‐dimethylamino‐ethyl)benzene]chromium tricarbonyl (**7**) and of the fluoro analogue (**8**) are reported in the CSD.^[35]^ They have quite different crystal packings, but they show short intermolecular Cl⋅⋅⋅O and F⋅⋅⋅O contacts (287.3 and 318.2 pm, the respective Nc values are 0.94 and 0.95) (Figure S9). We considered these derivatives are suitable models for comparing the HaB activation effect of Cr(CO)_3_ complexation on Cl and F atoms.

**Figure 4:**
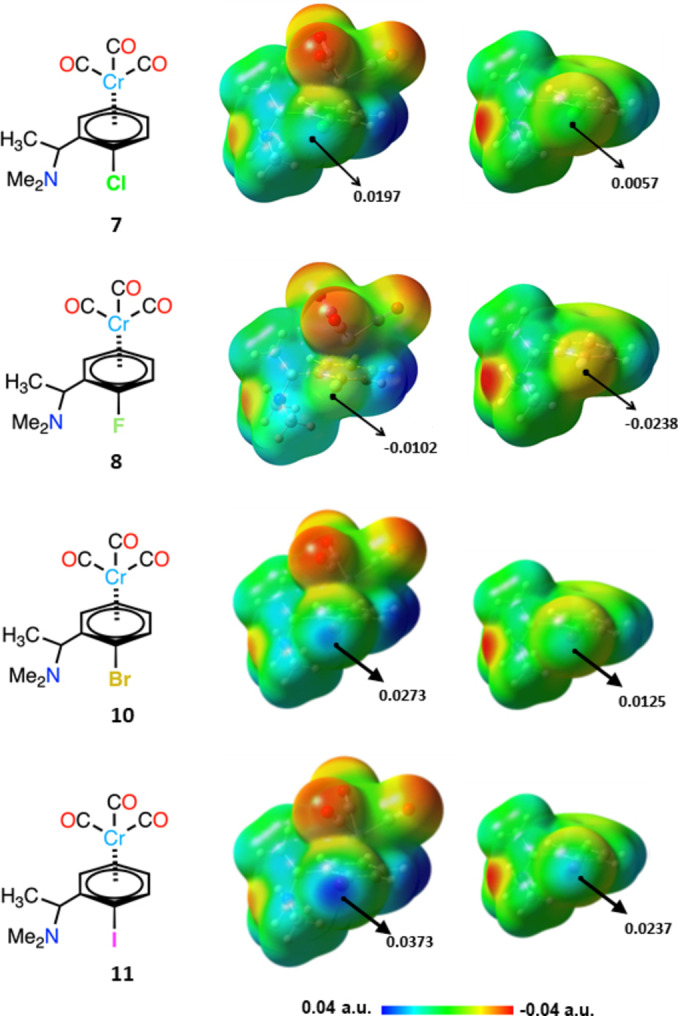
Structural formula (left) and MEP surfaces (mid, isovalue at density 0.001 a.u) for the η^6^‐Cr(CO)_3_ coordinated complexes **7**, **8**, **10** and **11** (a, b, c and d); MEP surfaces for corresponding non‐complexed arenes (right). In all MEPs, the N(CH_3_)_2_ groups are on the left and halogen atoms are in the front. Black dots indicate positions of maximum potential (values in a.u.). Geometries were optimized in the gas phase with relativistic (spin‐orbit) corrections (PW6B95‐D3‐DKH/def2‐QZVPPD), visualization – GaussView 6.0.16.

**Figure 5:**
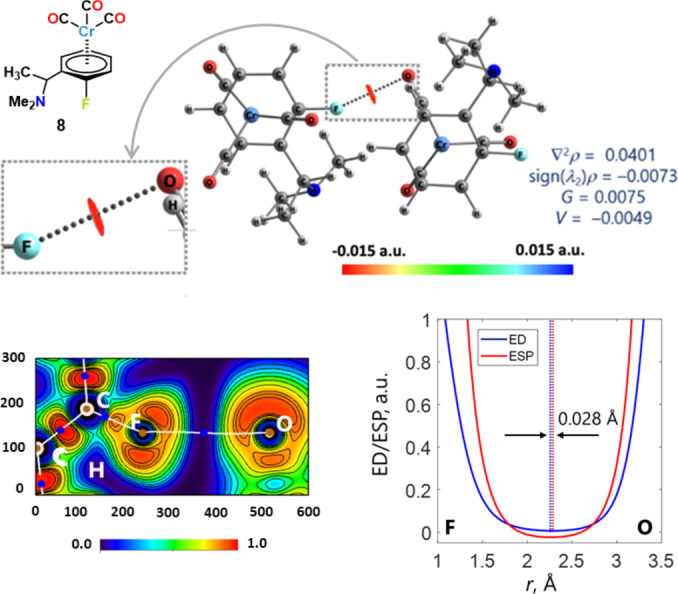
Top left: structural formula of complex **8**. Geometry of **8** dimer cut from the equilibrated unit cell. Top right: IRI isosurface (isovalue 1.0 a.u.) in the proximity of the F⋅⋅⋅O contact mapped by ED value, ED parameters in (3, −1) CPs of the F⋅⋅⋅O contact. The values of sign(λ_2_)ρ, Laplacian of electron density ∇^2^ρ, densities of local kinetic and potential electronic energies densities G and V (in blue, a.u.) confirm the weak attractive nature of the F⋅⋅⋅O interaction. Bottom left: Distribution of ELF in C−F⋅⋅⋅O plane of **8** dimers. Bond paths are white lines, electron density (3, −1) CPs are blue dots, (3, −3) CPs brown dots. Bottom right: Distribution of ED (blue) and ESP (red) along the F⋅⋅⋅O bond path for **8** dimer. Distance between ED and ESP minimum is given above the arrows.

The electrostatic potentials of the whole arenes (Cl and F atoms included) become more positive on Cr(CO)_3_ complexation (Figure 4a, 4b). In the chlorinated complex **7** the C−Cl⋅⋅⋅O contact is close to linearity (171.00°) and the potential computed at Cl σ‐hole is positive. This allows for the interaction to be dependably rationalized as an intermolecular HaB. Differently, in the fluorinated **8** complex the C−F⋅⋅⋅O angle (155.23°) shows non‐minor deviation from linearity and the electrostatic potential is negative on the whole F surface. Fluorine exceptionally acts as HaB donor[^36^, ^37^, ^38^, ^39^] and typically forms interactions wherein the C−F⋅⋅⋅nucleophile angle deviates significantly from linearity.^[2]^ Moreover, it is well known that atoms with a negative electrostatic potential on the 0.001 a.u. isosurface can act as σ‐hole bond donors thanks to polarization.[^40^, ^41^, ^42^, ^43^, ^44^] We nevertheless considered that additional computational studies were useful to reliably rationalize the F⋅⋅⋅O contact in **8** as a HaB. In order to obtain relaxed electron densities (EDs) which are more accurate than the “molecular” calculations performed with or without optimization,[^45^, ^46^] we used the “true” periodic calculations performed for unit cell by using VASP 6.0.3, GGA‐PBE‐PAW. The structure of the dimeric fragment of **8** and its IRI isosurface (1.0 a.u. isovalue) are shown in Figure 5. The red colour of IRI surface indicates the presence of an attractive noncovalent interaction between the F and O atoms. QTAIM analysis shows the presence of a (3; −1) CP along the line connecting F and O atoms. The calculated strength of the F⋅⋅⋅O interaction E_int_ is 1.8 kcal/mol, a fair value for a F centred HaB.^[2]^ The electron localization function (ELF) distribution (Figure 5, bottom left) demonstrates that the bond path is directed through the electron depleted region of the F atom, i. e. between ELF maxima regions (lone pairs) on F to the more electron rich region of the O atom. The philicity of the atoms in the F⋅⋅⋅O contacts in **8**, can be additionally revealed by analysing ED and electrostatic potential (ESP) distributions along the bond path (Figure 5, bottom right). The ED minimum is located closer to the F centre, while the ESP minimum is closer to the O centre (the distance between these minima is 2.8 pm). Based on the criterion proposed by Espinosa et al.,^[47]^ this allows for concluding that in the F⋅⋅⋅O HaB the F and O atoms function as the electrophile and nucleophile, respectively.

Analogous computational studies proved that the short F⋅⋅⋅O contacts in crystalline (*R*,*R*)‐(η^6^‐(*t*‐butyl(2‐ fluorophenyl)methylphosphine)(trihydrido)boron)‐chromium tricarbonyl^[48]^ (**9**, Figures S10, S11) are attractive HaB despite the non‐minor deviation from linearity.

The ability of Cr(CO)_3_ complexation to boost the σ‐hole donor propensity of elements as reluctant as fluorine being established, we assessed the analogous increase for other elements. For bromine and iodine, the two remaining halogen atoms, this has been done by considering the complexes analogous to the chloroarene‐Cr(CO)_3_ derivative **7** (compounds **10** and **11**, Figure 4c, 4d). As expected, in both Cr(CO)_3_ adducts the electrostatic potential on the whole molecule is more positive than in the uncomplexed arene moieties; the increase being of 0.0148 and 0.0136 a.u. at the σ‐hole opposite to the arene on Br and I, respectively.

**Figure 6:**
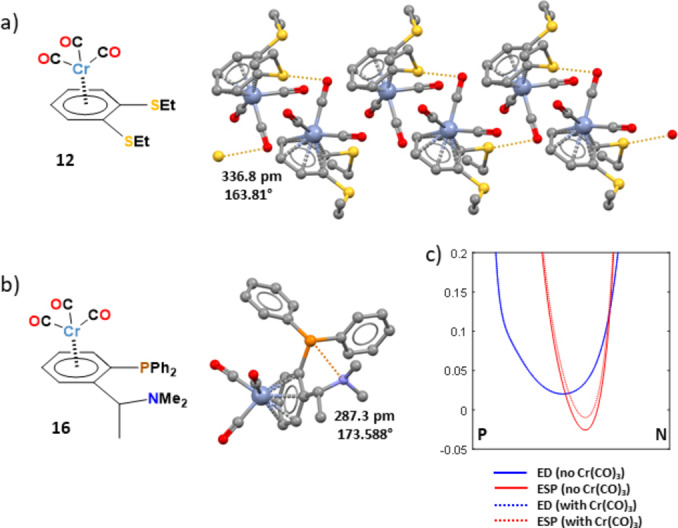
Structural formulas (left) and partial views of the crystal packing (ball and stick representation, Mercury) showing: a) an infinite chain of **12** (Refcode LEYHAA) assembled via ChB (ocher dotted lines); b) a molecule of **16** (Refcode SERFEA) whose conformation is locked by PnB (brownish dotted lines). S⋅⋅⋅O and P⋅⋅⋅N separations (pm) and C_Ar_−S⋅⋅⋅O and C_Ar‐Cr(CO)3_−P⋅⋅⋅N angles (°) are reported close to the interactions. Color coding: grey, carbon; red, oxygen; sky blue, chromium; ocher, sulfur; brownish, phosphorous; blue, nitrogen. c): Distribution of ED (blue) and ESP (red) along the P⋅⋅⋅N bond path for the Cr(CO)_3_‐arene derivative **16** and the uncomplexed phosphane moiety.

A CSD search showed that also groups 16, 15, and 14 elements when appended to η^6^‐arene‐Cr(CO)_3_ scaffolds form, with lone pair possessing atoms, short contacts which can be dependably rationalized as chalcogen,^[49]^ pnictogen,^[50]^ and tetrel^[51]^ bonds thanks to their geometry. For instance, crystals of the Cr(CO)_3_ derivative of 1,2‐bis(ethylthio)benzene^[52]^ (**12**) show the presence of chalcogen bonded supramolecular chains assembled by intermolecular C_Ar_−S⋅⋅⋅O contacts wherein the S⋅⋅⋅O distance and the C_Ar_−S⋅⋅⋅O angle are 321.8 pm (Nc=0.97) and 175.73° (Figure 6a). Calculation show that, similar to the halogen substituted η^6^‐Cr(CO)_3_‐arene derivatives discussed above, when Cr(CO)_3_ is coordinated, the electrostatic potential increases on the whole surfaces around the arene moiety (Figure S12), the increase being 0.013 a.u opposite to the C_Ar_−S bond. Similar MEP changes are shown by the η^6^‐arene‐Cr(CO)_3_ derivatives **13** and **14** wherein Se and Te substitute for S in the ring pendants of complex **12**. On the adopted MEP surface (isovalue of electron density 0.001 a.u.) the potential at the σ‐holes in **12**–**14** remains slightly negative. It is well known this does not prevent the site to form attractive σ‐hole interactions with donors of electron density, as, on interaction formation, polarization may induce favourable changes of the distribution of the electron density and at positions closer to the interaction distance, namely on smaller MEP surfaces, the potential will increase.^[40]^

Bis‐(η^6^‐chromium tricarbonyl)triphenylbismuthane^[53]^ **15** shows in the solid a short and intramolecular C_Ar‐Cr(CO)3_−Bi⋅⋅⋅O PnB (Nc=0.90) (Figure S13). Nitrogen is usually a better σ‐hole bonds acceptor than oxygen and in the amino‐phosphane derivative **16** the intramolecular C_Ar‐Cr(CO)3_−P⋅⋅⋅N PnB is particularly short (Nc=0.85)^[54]^ (Figure 6b). This interaction separation prevents a useful analyses of MEP surface of **16**. The ED and ESP distribution was considered along the P⋅⋅⋅N bond path revealing that, similar to halogen and chalcogen substituted arene‐Cr(CO)_3_ derivatives, the electrostatic potential increases on Cr(CO)_3_ coordination (Figure 6c).

The localization of the ED minimum closer to the P centre and of the ESP minimum closer to the N centre allows for establishing^[47]^ that in the P⋅⋅⋅N PnB the P is the electrophile and the N is the nucleophile.

**Figure 7:**
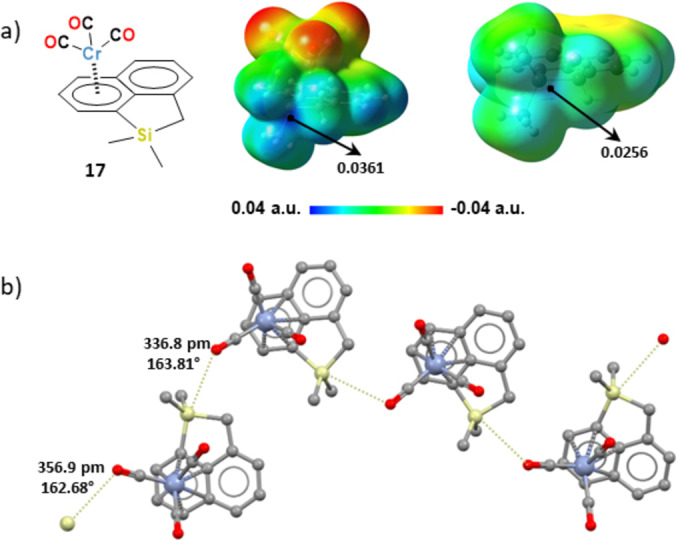
a) Structural formula (left) and MEP surface (isovalue at density 0.001 a.u.) for the η^6^‐Cr(CO)_3_ coordinated silaacenaphthylene **17** (mid) and the uncomplexed arene moiety (right). Black dots indicate positions of maximum potential (values in a.u.). b) Ball and stick representation (Mercury) of the infinite chain assembled in crystals of complex **17** (Refcode DIFCAW) via tetrel bonds (yellowish dotted lines). Si⋅⋅⋅O separations (pm) and C−Si⋅⋅⋅O angles (°) are reported close to the interactions. Color coding: grey, carbon; red, oxygen; sky blue, chromium; yellowish, silicon.

The 1‐silaacenaphthylene derivative^[55]^ **17** shows, in the solid, tetrel bonded infinite chains assembled by intermolecular Si⋅⋅⋅O contacts (the Si⋅⋅⋅O separations are 335.8 and 356.9 pm, Nc=0.92 and 0.98; Figure 7b). Here too, MEP calculations reveal that the electrostatic potential at the σ‐hole opposite to the arene has become more positive on Cr(CO)_3_ complexation, specifically, the increase was of 0.0105 a.u. (Figure 7a). In complexes **12**, **15**–**17** the σ‐hole interaction develops opposite to the covalent bond to the η^6^‐coordinated arene, suggesting that this may be the position of the most positive σ‐hole, namely that η^6^‐coordination of Cr(CO)_3_ units has played a specific role in promoting the atom electrophilicity.

## Conclusions

In conclusion, a library of η^6^‐Cr(CO)_3_‐coordinated chloroarenes was successfully synthesized and structurally characterized. Single‐crystal X‐ray diffraction analyses revealed the exceptional σ‐hole donor ability of the chlorine atoms, evidenced by the presence of short and highly directional Cl⋅⋅⋅O halogen bonds, even in weak donors such as chloroanisole. Electrostatic surface potential maps demonstrated a significant enhancement in the σ‐hole depth at the chlorine atom upon η^6^‐Cr(CO)_3_ coordination, with a sixfold increase in V_S,max_ values, from 0.003 a.u. in chloroanisole to 0.018 a.u. in η^6^‐Cr(CO)_3_‐chloroanisole.

Computational analyses using the Interaction Region Indicator (IRI) and Quantum Theory of Atoms in Molecules (QTAIM) confirmed the attractive nature of the observed Cl⋅⋅⋅O interactions. Furthermore, analogous computational studies on CSD structures of arene derivatives bearing halogen atoms or elements of groups 16, 15, and 14 covalently bonded to the arene moiety proved the generality of the increase of σ‐hole bond donor ability of the heteroatomic substituents when Cr(CO)_3_ is coordinated to the arene.

η^6^‐Coordination of the neutral Cr(CO)_3_ moiety is thus established as a versatile and reliable strategy for enhancing the σ‐hole donor ability of heteroatomic substituents on arenes. These findings offer new methodologies for modulating σ‐hole interactions, with the possibility of various applications, e. g. in reactivity and catalysis.

## Experimental Section

**Materials and Methods**: Chromium‐hexacarbonyl, *n*‐butyl ether, tetrahydrofuran (THF), dioxane, diglyme, *n*‐hexane, and chloroarenes were purchased from commercial suppliers (abcr and Sigma‐Aldrich) and used without further purification.

^1^H‐NMR spectra were recorded at ambient temperature on Nuclear Magnetic Resonance Spectrometer AVANCE III, Bruker‐BioSpin. All the chemical shifts are given in ppm and the Js in Hz. CD_3_COCD_3_ was used as both solvent and internal standard in NMR spectra. FT‐IR spectra were obtained using a Nicolet Nexus FT‐IR spectrometer equipped with UATR unit.

The single crystal data were collected using a XtaLAB Synergy diffractometer, equipped with a HyPix detector. Unit cell refinement and data reduction were performed using CrysAlisPro 1.171.41.98a. Structures were solved by direct methods using SHELXT and refined by full‐matrix least‐squares on F^2^ with anisotropic displacement parameters for the non‐H atoms using Olex2. Absorption correction was performed based on multi‐scan procedure.[^56^, ^57^, ^58^]

The geometries of η^6^‐chloroarene‐Cr(CO)_3_ complexes **1**–**6** were optimized and checked on the absence of imaginary vibrational harmonic frequencies at PW6B95^[59]^‐D3^[60]^/jorge‐TZP‐DKH^[61]^ level of theory using Gaussian 16 C.01 software package. The one‐component scalar (spin‐free) relativistic Hamiltonian, constructed with 2^nd^ order Douglass‐Kroll‐Hess series of unitary transformations of four‐component Dirac‐Coulomb Hamiltonian with neglecting of spin operators, was used to solve the electronic problem. The QTAIM analysis, IRI plots,^[62]^ ED and MEP^[63]^ distributions has been calculated by using the Multiwfn program (version 3.8).^[64]^ Calculations results were visualized using GaussView 6.0 program.

**General synthesis and procedure**: The compounds **1**–**6** were prepared by following a reported method.^[65]^ Cr(CO)_6_ (1 eq., 3.4 mmol) and the chloroarene (4 eq., 13.6 mmol) were added to a mixture of THF:*n*‐butyl ether (1 : 9, v/v) solution and refluxed for 48 h; alternatively dioxane/diglyme solvent mixtures were used. The reaction mixture was cooled to room temperature and filtered to remove the excess Cr(CO)_6_. Thus, obtained yellow solution was evaporated under reduced pressure using rotary evaporator. The yellow crude was dissolved in *n*‐hexane and filtered. The obtained clear solution was left to slowly evaporate, resulting into a yellow crystalline solid (yield 20–25 %). The synthesized compounds were stored in the dark and in a dry place.

**1**: Prismatic shaped yellowish crystals of compound **1** suitable for single crystal X‐Ray diffraction were obtained from slow evaporation of an *n‐*hexane solution. FT‐IR (selected peaks, cm^−1^) 3102, 1953, 1846, 1498, 1443, 1427, 1405, 1389, 1147, 1112, 1082, 1013, 814, 703, 661, 620, 533, 470. ^1^H NMR (400.13 MHz, CD_3_COCD_3_) δ 5.82 (m, 2H), 5.62 (m, 2H).

**2**: Prismatic shaped yellowish crystals of compound **2** suitable for single crystal X‐Ray diffraction were obtained from slow evaporation of an *n*‐hexane solution. FT‐IR (selected bands, cm^−1^) 3095, 2924, 1962, 1857, 1485, 1422, 1363, 1260, 1118, 1061, 994, 823, 777, 652, 612, 533, 483. ^1^H NMR (400.13 MHz, CD_3_COCD_3_) δ 6.12 (brs, 1H), 6.02 (t, J=6.5 Hz, 1H), 5.66 (dd, J=6.6, 1.7 Hz, 2H).

**3**: Block shaped yellowish crystals of compound **3** suitable for single crystal X‐Ray diffraction were obtained from slow evaporation of an *n*‐hexane solution. FT‐IR (selected bands, cm^−1^) 3101, 2924, 1961, 1862, 1493, 1443, 1345, 1090, 1010, 835, 809, 703, 652, 611, 532, 462. ^1^H NMR (400.13 MHz, CD_3_COCD_3_) δ 6.02 (s, 4H).

**4**: Plate shaped yellowish crystals of compound **4** suitable for single crystal X‐Ray diffraction were obtained from slow evaporation of an *n*‐hexane solution. FT‐IR (selected bands, cm^−1^) 3085, 2962, 1955, 1859, 1418, 1357, 1258, 1170, 1088, 1011, 794, 659, 621, 524, 468. ^1^H NMR (400.13 MHz, CD_3_COCD_3_) δ 5.96 (d, J=6.6 Hz, 1H), 5.73 (m, 1H), 5.61 (d, J=6.4 Hz, 1H), 2.47 (s, 3H).

**5**: Prismatic shaped yellowish crystals of compound **5** suitable for single crystal X‐Ray diffraction were obtained from overnight evaporation of an *n*‐hexane solution. FT‐IR (selected bands, cm^−1^) 3091, 2962, 1952, 1851, 1446, 1378, 1258, 1103, 1068, 1005, 802, 763, 659, 622, 526, 468. ^1^H NMR (400.13 MHz, CD_3_COCD_3_) δ 5.95 (d, J=7.7 Hz, 2H), 5.69 (m, 1H), 2.51 (s, 3H).

**6**: Block shaped yellowish crystals of compound 6 suitable for single crystal X‐Ray diffraction were obtained from overnight evaporation of an *n*‐hexane solution. FT‐IR (selected bands, cm^−1^) 3102, 2922, 1951, 1843, 1528, 1461, 1253, 1096, 1018, 796, 643, 615, 536, 472. ^1^H NMR (400.13 MHz, CD_3_COCD_3_) δ 6.08 (d, J=7.1 Hz, 2H), 5.57 (d, J=7.0 Hz, 2H), 3.74 (s, 3H).

## Conflict of Interests

The authors declare no conflict of interest.

1

## Supporting information

As a service to our authors and readers, this journal provides supporting information supplied by the authors. Such materials are peer reviewed and may be re‐organized for online delivery, but are not copy‐edited or typeset. Technical support issues arising from supporting information (other than missing files) should be addressed to the authors.

Supporting Information

## Data Availability

The data that support the findings of this study are available in the supplementary material of this article.
