# Supplementary material for: Upgrade of Weak σ‐Hole Bond Donors via Cr(CO)3 Complexation
Source: Chemistry. 2025 Jan 28;31(12):e202404570. doi: 10.1002/chem.202404570 (PMC11855244; doi:10.1002/chem.202404570)
Supplement: Supplementary file 1 — Supporting Information [file CHEM-31-e202404570-s001.pdf]

Chemistry–A European Journal

Supporting Information

Upgrade of Weak σ-Hole Bond Donors via $Cr(CO)_3$ Complexation

Arun Dhaka, Roberta Beccaria, Andrea Pizzi, Elena Yu. Tupikina, Vadim Yu. Kukushkin, and Giuseppe Resnati*

Supplementary Information

Table Of Content:

S1. NMR Spectra……………………………………..…………………………………..................3

S2. Cambridge Structural Database search…………………………..………..………...………...….7

S3. Crystallographic details for compounds 1-6……………………………………………………..…10

S4. Computational analyses of chloro- and fluoroarene-Cr(CO)3 complexes 1-9 and of complexes 12-15……………………………………………………...……………………………..19

S1. NMR Spectra

Fig. S1. Plots of ^{1}H NMR spectra of η^{6}-chloroarene-$Cr(CO)_3$ complexes. Peaks of impurities and solvents are marked with an asterisk.

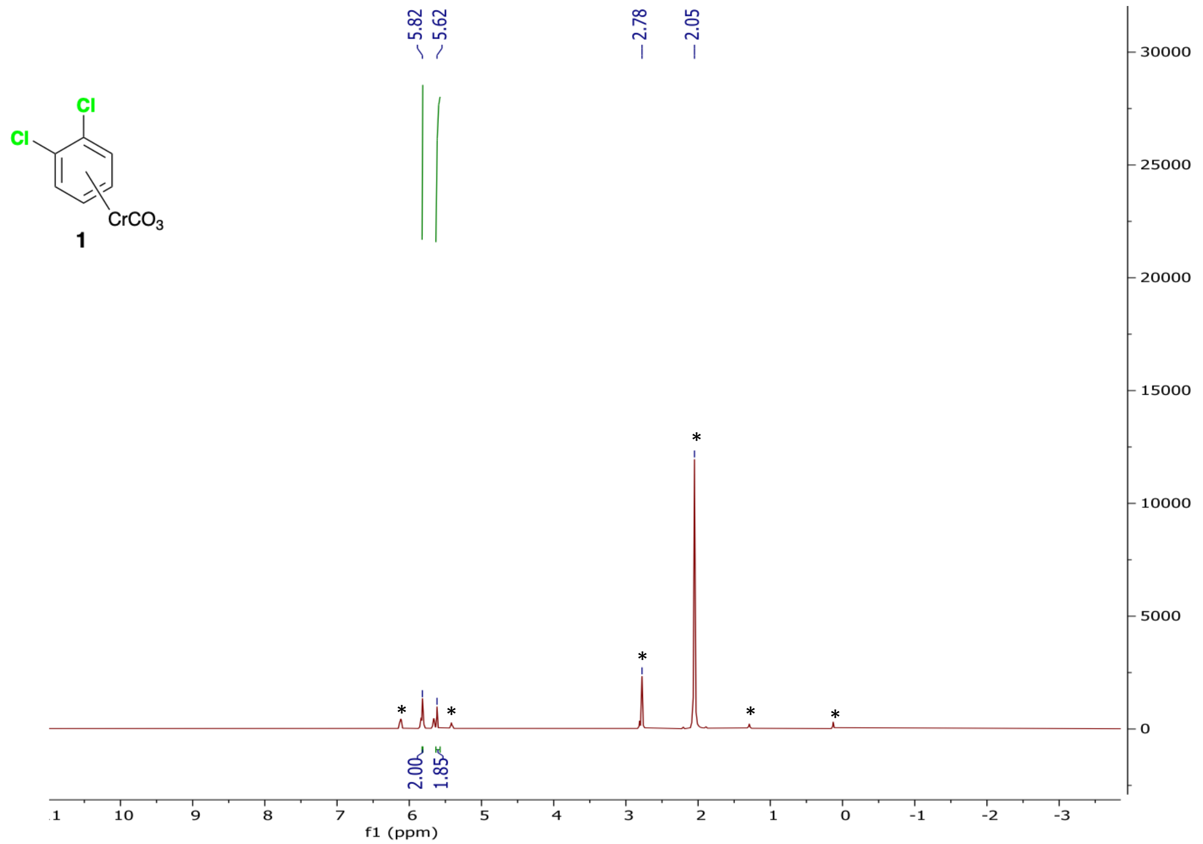

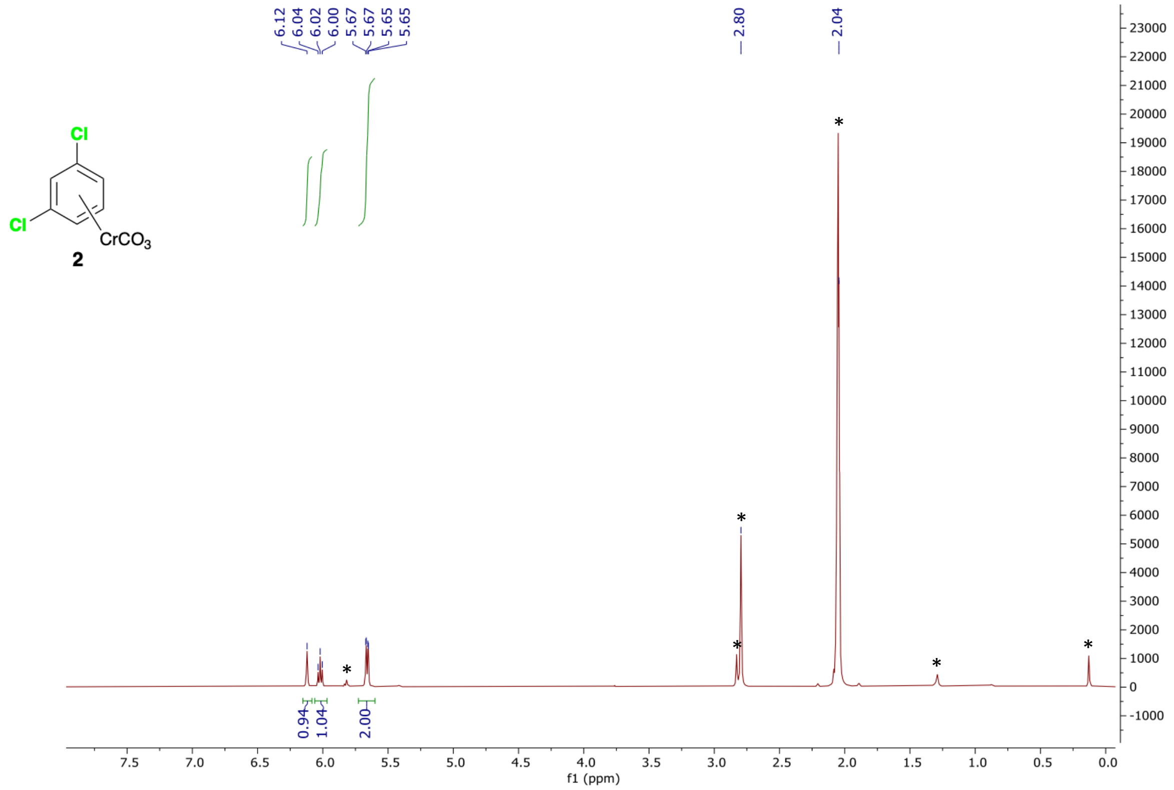
6.12
6.04
6.02
6.00
5.67
5.67
5.65
5.65
2.80
2.04
Cl
Cl
CrCO3
2
0.94
1.04
2.00
*
f1 (ppm)

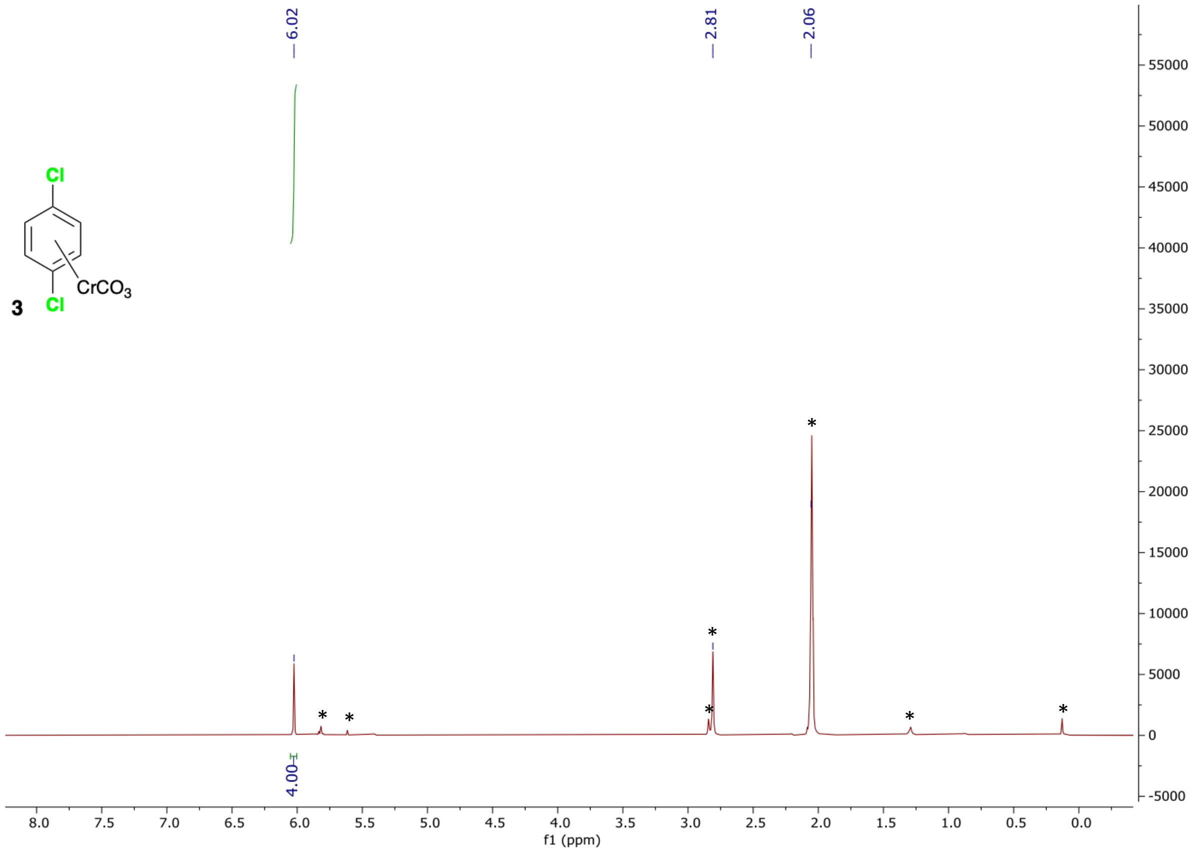
6.02
2.81
2.06
Cl
Cl
CrCO3
3
4.00
*
f1 (ppm)

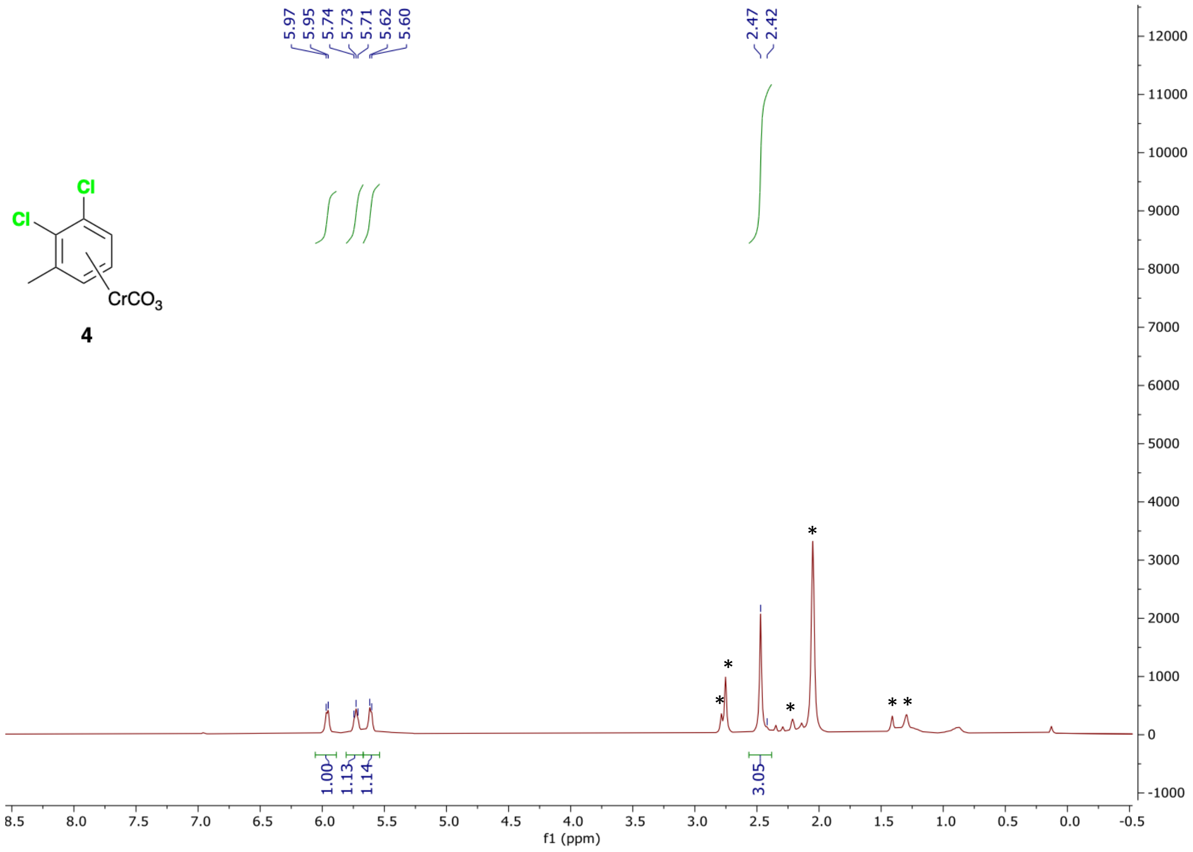
Cl
Cl
CrCO3
4
5.97
5.95
5.74
5.73
5.71
5.62
5.60
2.47
2.42
1.00
1.13
1.14
3.05
f1 (ppm)

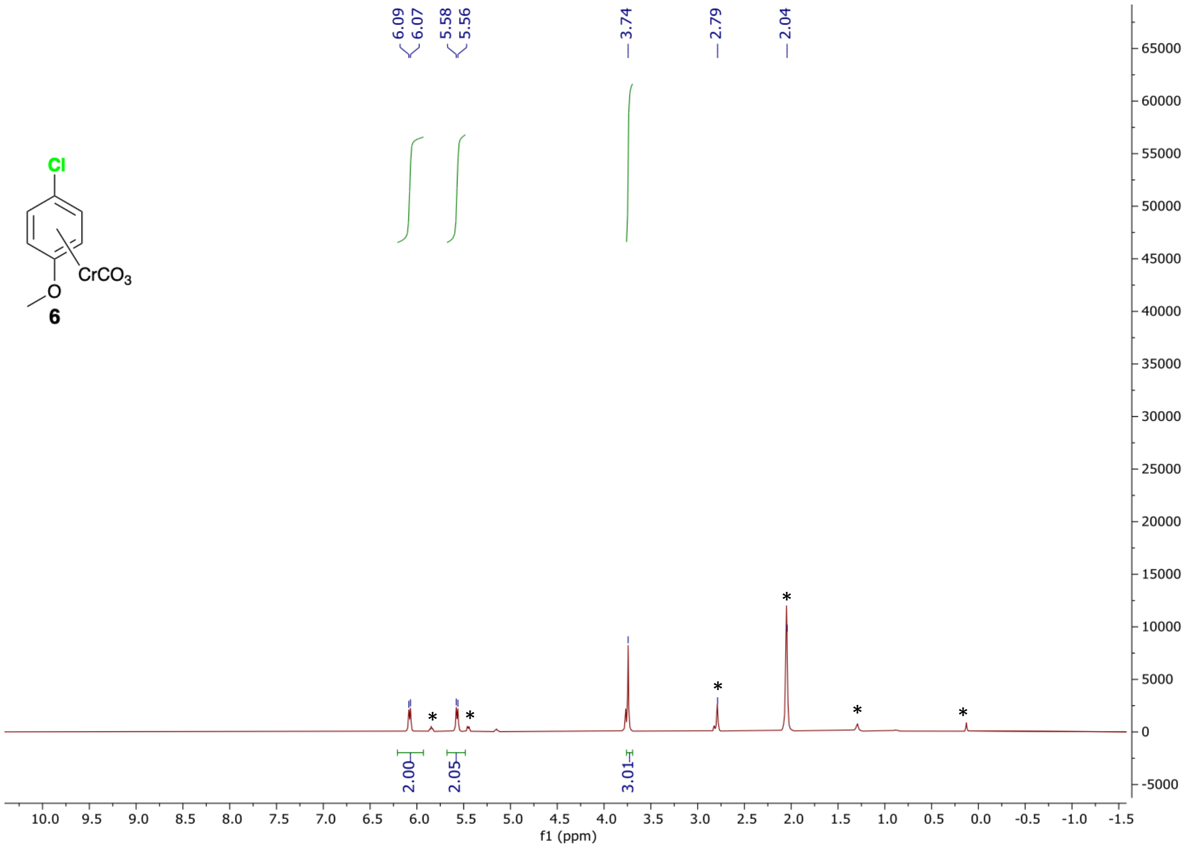
Cl
CrCO3
O
6
6.09
6.07
5.58
5.56
3.74
2.79
2.04
2.00
2.05
3.01
f1 (ppm)

S2. Cambridge Structural Database search

Table S1. A Cambridge Structural Database search (CSD 2024.1.0 version) for crystal structures containing at least one oxygen atom and one dichloro-benzene unit (*ortho*-, or *meta*-, or *para*-dichlorobenzene) identified 340 structures, the respective Refcodes are given below. Only 14 structures (Refcodes in blue), i.e. approximately 4.1%, show a Cl···O halogen bond (Cl· ··O distance ≤ 335 pm, namely shorter than the sum of van der Waals radii, C-Cl···O angle ≥ 160°).

ABUREY	ACOTES	AFOXEY	AFOXEY01	AGAVOW	AGIGEF	AJIPEP	AMAGEA		AMAGEA01	APUREJ
ASAROC	ASOGEW	ATOZUH	ATUBAV	AXOPEL	AXOPIP	BAKPAF	BASFOR		BAVLUI	BAVVAV
BODPDI	BUGXOT	CABHIA	CEDMIK	CELXOH	CETGIR	COCDUU	COVBAR		DAQMUD	DEHVEU
DIBHON	DOBQUJ	DORQIK	DORQIK01	DOVVOC	DOZXIC	DOZXOI	DUKBES		DUTFEG	EBUZAE
EBUZAE01	ECOPOD	EFIVIB	EGAGOI	EGEWAP	EJAQOV	EKOZOT	ENIYAC		ENIYIK	EQICIS
ERADEI	ESODAT	ESUMOV	ESUMOV01	ETUHIK	EWIHID	FAQTEZ	FEBREL		FEFQOY	FIFGEH
FINDUD	FIRROQ	FOLFIW	FOVWOE	FOYCON	FOYDAA	FUNNOT	FUTSEV		GAKRIX	GALGOR
GAMSUK	GECZUM	GEDBEZ	GEDBID	GIBVIZ	GINVEG	GOSYOC	GOXKAI		GUNDIE	GUSRET
GUXXIJ	HASJEU	HASKAR	HEJPAN	HEYHUR	HIDSAQ	HIDTIZ	HODKET		HODKIX	HODKUJ
HUPHIM	HUPHOS	HUPJIO	HUPNEO	HURHUZ	IBEHUV	IBEJOR	IFONAT		IFOXOQ	IGIZOO
IGIZOO01	IMAKAJ	IMAKEN	IPAKAO	IQENIE	IQONAG	IQONEK	ITOKEJ		ITOREQ	IXAVEL
JAHBOM	JOGRAB	JUNQIV	JUYHAP	JUYHET	KABZIZ	KADXEU	KAHHUX		KAMPOH	KASLIB
KAVDOB	KAVDOB01	KIDMIU	KIPZUG	KIVBAV	KIVBEZ	KIVBID	KOXJEP		KOXJIT	KUWBEM
KUWBIQ	KUYHAN	LAFVUM	LAFWAT	LAFWIB	LAFWOH	LAFWUN	LAFXIC		LALRIZ	LAQZIO
LIFBAD	LIHREZ	LOPKUW	LUKMOU	MAQGIY	MAQSAY	MATBAO	MATBES		MATBIW	MEDKIS
MEDNER	MERHUP	MERJUR	MERKAY	MERPUU	METMON	METNEE	MIWLEM		MODEPH	MUDQUY
NACTIW	NAGPEU	NAGSAT	NAGSOH	NEZQIU	NIRCAV	NIRCID	NOJPOR		NUHZAT	OCOJEX
OCUMAD	OCUMEH	ODURIP	OFASOF	OFOGOI	OHAGUA	OMICEU	OMIRIO		ORAGEV	ORAGIZ
ORAGUL	ORUNIB	OWUMUQ	OWUNEB	OXADIB	OYAVAO	OZAJIL	OZAJOR		PACVOF	PACWUM
PACXAT	PAYKAE	PAYKEI	PAYKIM	PAYRIT	PAYROZ	PEHLET	PERNOP		PERNOP01	PIVCRW
PODKAX	POLYEX	PONMEL	POQJOU	POQJOU01	PUJROB	PUZCUJ	PUZDAQ		PUZDEU	PUZDUK
PUZFAS	PUZFEW	PUZFIA	PUZFOG	PUZFUM	QAJBEL	QAJBIP	QASTIP		QEGGUE	QICKIX
QIVWEA	QOBWOT	QOHZUK	QOJBEY	QOJBIC	QOJBOI	QOWLUM	QUJFUW		QUMVAY	QUSFUF
RAQGAS	REBLAL	REJVUZ	REJWEK	RENMUV	REPHUS	REVJEK	RIRVIX		RIZWIH	RIZWON
ROBHEW	RONCIJ	SEHRUU	SEHSAB	SEZQUL	SIWKAN	SIWKER	SIYSUQ		SOLRET	SUBGON
SUPCEN	TEBZIM	TOQJUF	TOXRAC	TOYFIZ	TOYFOF	TPSNRE	TUXKAB		UGUQOD	UHUVEZ
UHUVID	UJOXOH	UJOXUN	UKOVAU	USUSAF	UWOHIA	VANHUR	VEPGON		VIHRIQ	VIHROW
VIPBUU	VIPCAB	VIZNUP	VIZPOK	VOBKUV	VOBLAC	VOCCEY	VOSJEV		VOSKAS	VOVBIU
VOVBOA	VOVBUG	VOWCUF	VUSYEN	VUSYIR	VUTBOC	WAJJIE	WEJJEA		WEJJEA01	WENJAD
WIJMOS	WODKIM	WODKOS	WODKUY	WODLAF	WODLEJ	WODLIN	WODMEJ		WOFJAD	WUYLAD
XANKAB	XANYAP	XAQWAN	XAQWUH	XAQXIW	XEFNED	XEFYUE	XIJQIQ		XIJQIQ01	XIJQUD
XOKFEK	XUQMEE	XUQMII	XUQMOO	YAPWOE	YAPWOE	YAPWUK	YAPWUK		YEKDOK	YEXCIQ
YONZIN	YONZOT	YOTMIF	YUQLUU	YUQMEF	YUQMOP	YUQMUV	YUQNAC		YUQNEG	ZARXUO
ZESGEL	ZEZKIY	ZIPSUP	ZIPSUP01	ZITMUK	ZUGVII	ZUGVOO	ZUNNUU		ZUNPAC	ZUNPIK

Fig. S2. Scatterplot of the Cl···O distance (Å) vs. the C-Cl···O angle (°) of CSD structures containing an oxygen atom and 1,2-Cl_2-C_6H_4 or the 1,3- and 1,4 isomers and wherein the Cl···O distance is in between 3.0 and 3.9 Å and the C-Cl···O angle is in between 130° and 180°. Refcodes of the structures and respective Cl···O distance and C-Cl···O angle are listed under the scatterplot.

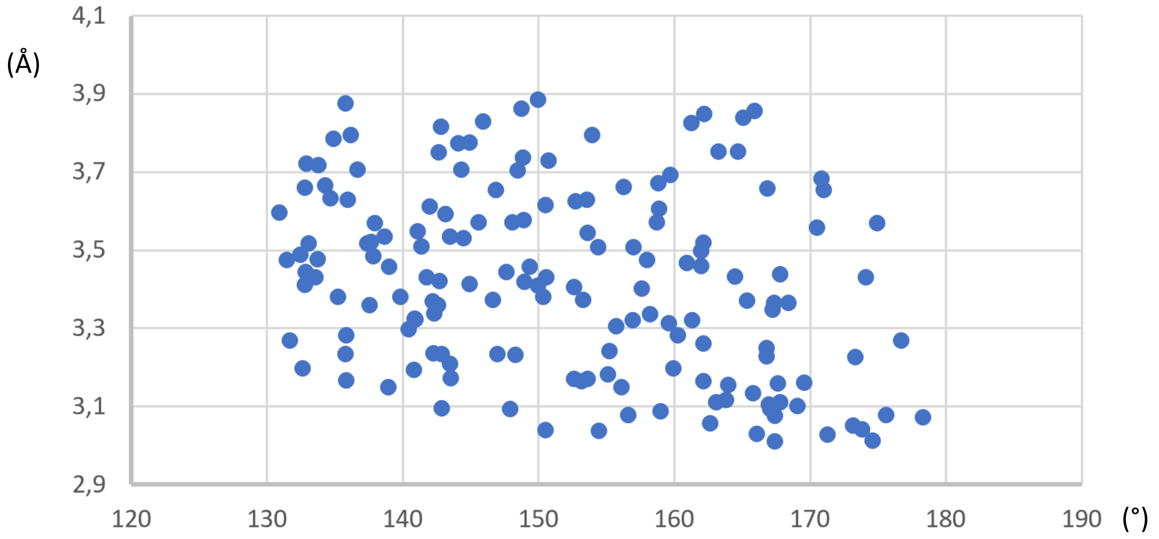

1,2-Cl_2-C_6H_4								
Refcode	***ANG (°)***	***DIST. (Å)***	***Refcode***	***ANG (°)***	***DIST. (Å)***	***Refcode***	***ANG (°)***	***DIST. (Å)***
MOCFET	175,639	3,078	HUPHOS	166,794	3,227	REFHUJ	135,768	3,874
QIVHEM	165,379	3,37	HUPHOS	156,125	3,148	REFJAR	161,315	3,32
QIVHEM	140,96	3,321	IBEJOR	144,463	3,529	REFJAR	166,853	3,657
SODTEO	166,821	3,248	IQONEK	147,01	3,233	REFJAR	162,163	3,164
ACOTES	145,594	3,571	JIBJUD	142,594	3,358	REJVUZ	135,954	3,628
ACOTES	150,544	3,615	JUYHAP	134,928	3,785	RENMUV	169,11	3,101
AFOXEY	132,854	3,444	JUYHAP	148,982	3,418	RIPSOC	159,005	3,086
AFUVEG	173,869	3,041	JUYHET	142,855	3,816	RIPSUI	159,594	3,313
AFUVEG	173,19	3,05	JUYHET	140,825	3,193	RIPTOD	176,718	3,268
AFUVEG	143,558	3,172	KIDMIU	158,016	3,474	RIPTOD	132,636	3,196
AFUVEG	142,872	3,234	KIVBAV	163,097	3,11	RIPVAR	152,633	3,404
ATOZUH	164,473	3,431	KIVBEZ	140,469	3,296	RIPVAR	149,957	3,409
ATOZUH	149,95	3,884	LASXEM	167,433	3,075	RIPVIZ	150,531	3,039
ATOZUH	159,965	3,196	MAQSAY	174,978	3,568	RIPVIZ	143,477	3,209
ATOZUH	158,853	3,671	MATBAO	155,111	3,182	RISXEA	133,056	3,516
ATOZUH	148,071	3,57	MATBES	148,303	3,232	RISXEA	160,281	3,281
ATOZUH	157,018	3,507	MEDKIS	158,901	3,606	RISXEA	140,864	3,323
CIBWET	135,247	3,38	MEDNER	158,737	3,57	RITCAC	171,301	3,027
CIBWET	178,323	3,071	MERPUU	154,409	3,506	RONCIJ	144,932	3,774
CIBWET	141,75	3,429	MERPUU	168,421	3,364	RONCIJ	156,614	3,078
CIBWET01	142,293	3,236	MIPRAI	167,441	3,009	SIYSUQ	137,668	3,52
CIBWET01	153,624	3,544	NACTIW	136,704	3,705	SIYSUQ	144,304	3,705
CIBWET01	139,859	3,379	NACTIW	174,126	3,429	SIYSUQ	154,484	3,037

CIBWET01	136,165	3,794	NAGSOH	133,799	3,717	SUBGON	146,661	3,371
CIBWET01	132,813	3,41	ORAGEV	133,568	3,429	TOYFIZ	141,093	3,547
CIBWET02	142,243	3,369	ORAGEV	162,177	3,261	TOYFOF	141,406	3,509
CIBWET02	138,996	3,457	ORAGIZ	153,95	3,794	UGUQOD	148,461	3,704
CIBWET02	132,804	3,659	ORAGUL	148,944	3,576	UGUQOD	135,828	3,282
CIBWET02	144,914	3,412	ORAGUL	137,976	3,569	UGUQOD	164,715	3,751
DEXHIB	161,285	3,824	OYAVAO	162,014	3,497	VIBQOQ	173,349	3,226
DIMJOC	174,604	3,012	PERNOP	152,734	3,625	VIPCAB	147,621	3,444
DOBQUJ	134,666	3,632	PERNOP	165,948	3,856	VIZNUP	138,926	3,148
DOBQUJ	170,847	3,683	PERNOP01	153,575	3,629	VIZNUP	132,495	3,488
DOBQUJ	143,488	3,534	PERNOP01	165,081	3,839	VOVBIU	167,36	3,365
DUTFEG	148,882	3,736	POLYEX	132,92	3,721	WAJJIE	152,642	3,17
EGEWAP	155,243	3,241	PUZFAS	167,833	3,109	WODKOS	170,52	3,556
ESUMOV	145,922	3,828	PUZFEW	166,974	3,105	WODKUY	131,476	3,475
ESUMOV	156,262	3,661	PUZFIA	158,24	3,336	WODKUY	169,558	3,16
EWIHID	155,757	3,305	PUZFIA	167,097	3,092	WODLAF	166,089	3,029
EWIHID	162,016	3,459	PUZFIA	142,638	3,75	WODLAF	156,93	3,32
EWIHID	135,818	3,233	PUZFIA	167,815	3,438	WODLEJ	146,891	3,653
EWIHID	147,921	3,092	PUZFOG	134,308	3,664	WODLIN	130,897	3,596
FOVWOE	157,602	3,401	PUZFOG	142,906	3,094	WODLIN	142,744	3,42
FOYCON	167,618	3,158	QASTIP	148,73	3,861	WODLIN	133,76	3,476
HEFVOH	163,29	3,752	QASTIP	150,335	3,379	WODLIN	144,083	3,772
HETQIK	162,643	3,056	QICKIX	167,237	3,347	XAQWUH	160,942	3,466
HINXIP	142,317	3,337	QUMVAY	153,159	3,163	YEJHII	143,148	3,591
HINZAJ	159,72	3,691	QUMVAY	137,558	3,359	YUQNAC	165,794	3,133
HINZAJ	153,307	3,371	QUMVAY	150,583	3,429	ZARXUO	164,014	3,155
HODKIX	135,873	3,165	QUMVAY	137,398	3,516	ZARXUO	150,749	3,728
HODKUJ	162,158	3,518	RAWDUS	163,833	3,116	ZARXUO	149,377	3,456
HODKUJ	131,676	3,267	REFHIX	153,618	3,169	ZUNNUU	162,197	3,848
**1,3-Cl_2-C_6H_4**								
Refcode	***ANG (°)***	***DIST. (Å)***	***Refcode***	***ANG (°)***	***DIST. (Å)***	***Refcode***	***ANG (°)***	***DIST. (Å)***
XECWOV	138,674	3,534						
**1,4-Cl_2-C_6H_4**								
Refcode	***ANG (°)***	***DIST. (Å)***	***Refcode***	***ANG (°)***	***DIST. (Å)***	***Refcode***	***ANG (°)***	***DIST. (Å)***
IFOXOQ	137,843	3,483	IFOXOQ	171,043	3,653	KAVDOB	141,997	3,61

S3. Crystallographic details for compounds 1-6

Table S2. Crystal data and structure refinement for compound **1**.

**Identification code**	**1**
**Empirical formula**	$C_9H_4Cl_2CrO_3$
**Formula weight**	283.02
**Temperature/K**	298.15
**Crystal system**	triclinic
**Space group**	P-1
**a/Å**	6.53160(10)
**b/Å**	7.31460(10)
**c/Å**	11.2095(2)
**α/°**	94.086(2)
**β/°**	96.623(2)
**γ/°**	105.570(2)
**Volume/Å^3**	509.518(15)
**Z**	2
**ρ_{calc}g/cm^3**	1.845
**μ/mm^{-1}**	13.936
**F(000)**	280.0
**Crystal size/mm^3**	0.2 × 0.1 × 0.05
**Radiation**	Cu Kα (λ = 1.54184)
**2Θ range for data collection/°**	7.986 to 152.722
**Index ranges**	-8 ≤ h ≤ 7, -8 ≤ k ≤ 9, -13 ≤ l ≤ 14
**Reflections collected**	18548
**Independent reflections**	1998 [R_{int} = 0.0461, R_{sigma} = 0.0190]
**Data/restraints/parameters**	1998/0/137
**Goodness-of-fit on F^2**	1.130
**Final R indexes [I>=2σ (I)]**	R_1 = 0.0610, wR_2 = 0.2095
**Final R indexes [all data]**	R_1 = 0.0641, wR_2 = 0.2137
**Largest diff. peak/hole / e Å^{-3}**	0.73/-1.03
**CCDC Number**	2370548

Table S3. Crystal data and structure refinement for compound **2**.

**Identification code**	**2**
**Empirical formula**	$C_9H_4Cl_2CrO_3$
**Formula weight**	283.02
**Temperature/K**	149.99(10)
**Crystal system**	monoclinic
**Space group**	C2
**a/Å**	12.3889(2)
**b/Å**	7.42470(10)
**c/Å**	10.8525(2)
**α/°**	90
**β/°**	96.8860(10)
**γ/°**	90
**Volume/Å^3**	991.05(3)
**Z**	4
**ρ_{calc}g/cm^3**	1.897
**μ/mm^{-1}**	14.330
**F(000)**	560.0
**Crystal size/mm^3**	0.2 × 0.15 × 0.02
**Radiation**	Cu Kα (λ = 1.54184)
**2Θ range for data collection/°**	8.206 to 152.354
**Index ranges**	-15 ≤ h ≤ 14, -8 ≤ k ≤ 9, -13 ≤ l ≤ 13
**Reflections collected**	9115
**Independent reflections**	1897 [R_{int} = 0.0478, R_{sigma} = 0.0288]
**Data/restraints/parameters**	1897/1/137
**Goodness-of-fit on F^2**	1.040
**Final R indexes [I>=2σ (I)]**	R_1 = 0.0315, wR_2 = 0.0845
**Final R indexes [all data]**	R_1 = 0.0316, wR_2 = 0.0847
**Largest diff. peak/hole / e Å^{-3}**	0.54/-0.61
**Flack parameter**	0.479(9)
**CCDC Number**	2370552

Table S4. Crystal data and structure refinement for compound **3**.

**Identification code**	**3**
**Empirical formula**	$C_9H_4Cl_2CrO_3$
**Formula weight**	283.02
**Temperature/K**	150
**Crystal system**	triclinic
**Space group**	P-1
**a/Å**	6.9844(3)
**b/Å**	7.3054(3)
**c/Å**	11.0470(5)
**α/°**	84.995(4)
**β/°**	89.075(4)
**γ/°**	65.809(4)
**Volume/Å^3**	512.08(4)
**Z**	2
**ρ_{calc}g/cm^3**	1.836
**μ/mm^{-1}**	13.866
**F(000)**	280.0
**Crystal size/mm^3**	0.3 × 0.2 × 0.05
**Radiation**	Cu Kα (λ = 1.54184)
**2Θ range for data collection/°**	8.036 to 153.456
**Index ranges**	-8 ≤ h ≤ 8, -8 ≤ k ≤ 8, 0 ≤ l ≤ 13
**Reflections collected**	2003
**Independent reflections**	2003 [R_{int} = ?, R_{sigma} = 0.0473]
**Data/restraints/parameters**	2003/204/256
**Goodness-of-fit on F^2**	1.070
**Final R indexes [I>=2σ (I)]**	R_1 = 0.0963, wR_2 = 0.2496
**Final R indexes [all data]**	R_1 = 0.1002, wR_2 = 0.2532
**Largest diff. peak/hole / e Å^{-3}**	1.44/-1.06
**CCDC Number**	2370545

In the complex **3**, both the dichlorobenzene unit and the $Cr(CO)_3$ unit show some disorder which has been modelled by splitting atoms over different positions with different occupancies.

Table S5. Crystal data and structure refinement for compound **4.**

**Identification code**	**4**
**Empirical formula**	$C_{10}H_6Cl_2CrO_3$
**Formula weight**	297.05
**Temperature/K**	226(100)
**Crystal system**	triclinic
**Space group**	P-1
**a/Å**	6.44120(10)
**b/Å**	7.22280(10)
**c/Å**	11.9348(3)
**α/°**	90.457(2)
**β/°**	90.333(2)
**γ/°**	103.6990(10)
**Volume/Å^3**	539.419(18)
**Z**	2
**ρ_{calc}g/cm^3**	1.829
**μ/mm^{-1}**	13.197
**F(000)**	296.0
**Crystal size/mm^3**	0.2 × 0.1 × 0.05
**Radiation**	Cu Kα (λ = 1.54184)
**2Θ range for data collection/°**	7.408 to 152.586
**Index ranges**	-8 ≤ h ≤ 8, -8 ≤ k ≤ 6, -14 ≤ l ≤ 14
**Reflections collected**	10012
**Independent reflections**	2115 [R_{int} = 0.0504, R_{sigma} = 0.0338]
**Data/restraints/parameters**	2115/1/164
**Goodness-of-fit on F^2**	1.081
**Final R indexes [I>=2σ (I)]**	R_1 = 0.0295, wR_2 = 0.0722
**Final R indexes [all data]**	R_1 = 0.0320, wR_2 = 0.0741
**Largest diff. peak/hole / e Å^{-3}**	0.32/-0.35
**CCDC Number**	2370556

In the complex **4**, the methyl and the 2-chloro substituents show some positional disorder which has been modelled by splitting the substituents over two positions.

Table S6. Crystal data and structure refinement for compound **5.**

**Identification code**	**5**
**Empirical formula**	$C_{10}H_6Cl_2CrO_3$
**Formula weight**	297.05
**Temperature/K**	149.99(10)
**Crystal system**	triclinic
**Space group**	P-1
**a/Å**	6.47040(10)
**b/Å**	7.28380(10)
**c/Å**	11.6774(3)
**α/°**	91.263(2)
**β/°**	91.797(2)
**γ/°**	104.459(2)
**Volume/Å^3**	532.398(18)
**Z**	2
**ρ_{calc}g/cm^3**	1.853
**μ/mm^{-1}**	13.372
**F(000)**	296.0
**Crystal size/mm^3**	0.2 × 0.1 × 0.05
**Radiation**	Cu Kα (λ = 1.54184)
**2Θ range for data collection/°**	7.578 to 153.24
**Index ranges**	-7 ≤ h ≤ 8, -8 ≤ k ≤ 9, -14 ≤ l ≤ 13
**Reflections collected**	18702
**Independent reflections**	2100 [R_{int} = 0.0803, R_{sigma} = 0.0333]
**Data/restraints/parameters**	2100/0/140
**Goodness-of-fit on F^2**	1.103
**Final R indexes [I>=2σ (I)]**	R_1 = 0.0983, wR_2 = 0.2962
**Final R indexes [all data]**	R_1 = 0.1070, wR_2 = 0.3043
**Largest diff. peak/hole / e Å^{-3}**	1.58/-1.80
**CCDC Number**	2370555

Table S7. Crystal data and structure refinement for compound **6**.

**Identification code**	**6**
**Empirical formula**	$C_{20}H_{14}Cl_2Cr_2O_8$
**Formula weight**	557.21
**Temperature/K**	149.93(13)
**Crystal system**	triclinic
**Space group**	P-1
**a/Å**	7.1619(2)
**b/Å**	12.0543(2)
**c/Å**	12.9878(2)
**α/°**	103.276(2)
**β/°**	102.548(2)
**γ/°**	90.299(2)
**Volume/Å^3**	1063.43(4)
**Z**	2
**ρ_{calc}g/cm^3**	1.740
**μ/mm^{-1}**	11.156
**F(000)**	560.0
**Crystal size/mm^3**	0.4 × 0.3 × 0.1
**Radiation**	Cu Kα (λ = 1.54184)
**2Θ range for data collection/°**	7.176 to 153.342
**Index ranges**	-8 ≤ h ≤ 9, -15 ≤ k ≤ 14, -15 ≤ l ≤ 16
**Reflections collected**	19478
**Independent reflections**	4241 [R_{int} = 0.0577, R_{sigma} = 0.0303]
**Data/restraints/parameters**	4241/28/323
**Goodness-of-fit on F^2**	1.063
**Final R indexes [I>=2σ (I)]**	R_1 = 0.0599, wR_2 = 0.1670
**Final R indexes [all data]**	R_1 = 0.0622, wR_2 = 0.1690
**Largest diff. peak/hole / e Å^{-3}**	1.48/-1.42
**CCDC Number**	2370553

In the complex **6**, one of the two crystallographically independent chloroanisole molecules show some disorder which has been modelled by splitting atoms over two positions.

Fig. S3. Partial view (ball and stick representation, Mercury) of the crystal packing of complexes **1** (top) and **2** (bottom). Cl···O halogen bonds are black dotted lines; H···Cl and H···O hydrogen bonds are grey dotted lines. Cl···O separations (pm) and C–Cl···O angles (°) are reported close to the interactions. Color coding: grey, carbon; whitish, hydrogen; red, oxygen; sky blue, chromium; green, chlorine.

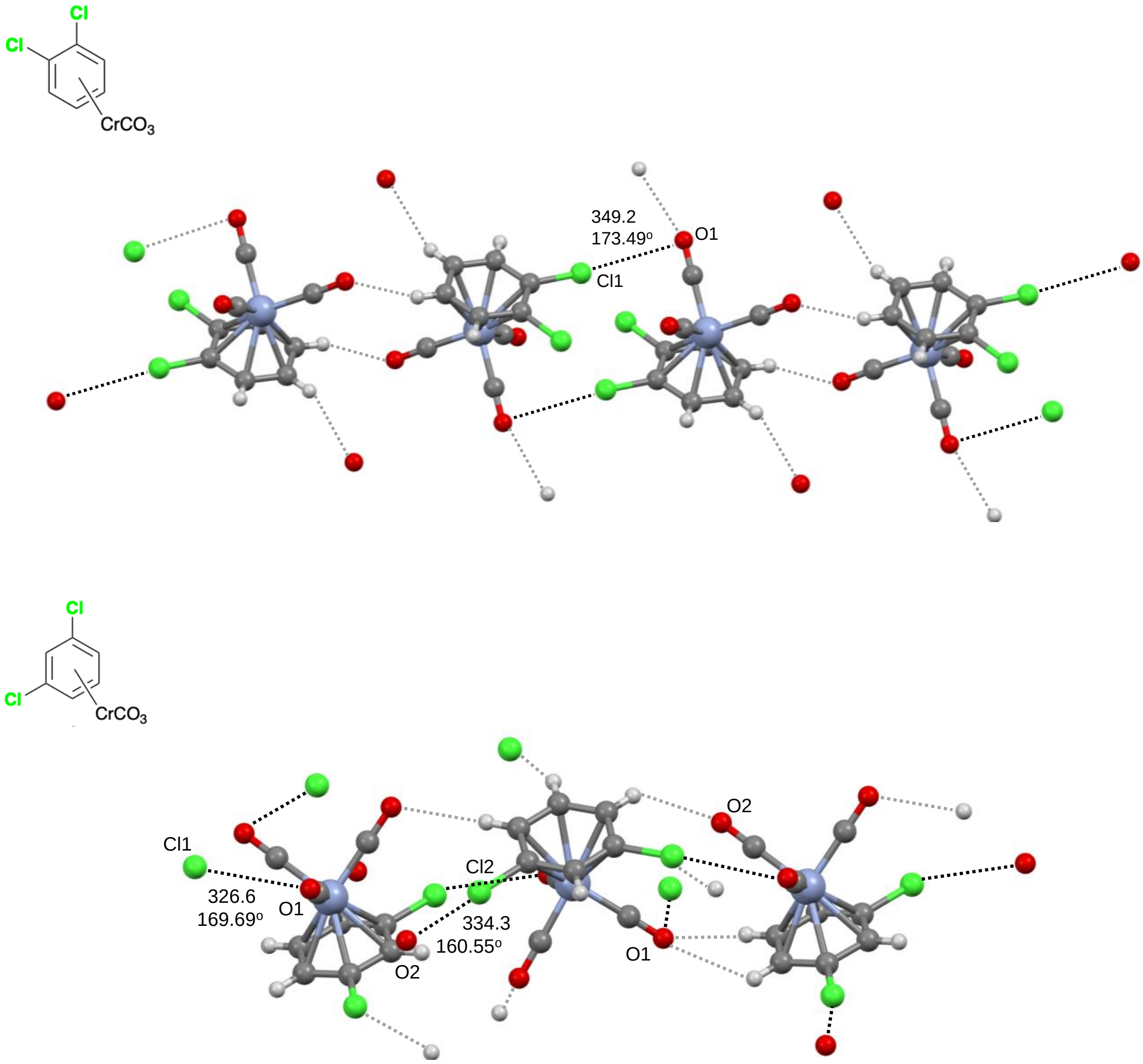

Fig. S4. Partial view (ball and stick representation, Mercury) of the crystal packing of complexes **3** (top) and **4** (bottom). In the complex **3**, only one occupancy of those used for modelling the disorder of both the dichlorobenzene unit and the $Cr(CO)_3$ unit has been reported in the figure for sake of clarity. In the complex **4**, only one occupancy of those used for modelling the disorder of the methyl and 3-chloro substituents has been reported in the figure for sake of clarity. Cl···O halogen bonds are black dotted lines; H···Cl and H···O hydrogen bonds are grey dotted lines. Cl···O separations (pm) and C–Cl···O angles (°) are reported close to the interactions. Color coding: grey, carbon; whitish, hydrogen; red, oxygen; sky blue, chromium; green, chlorine.

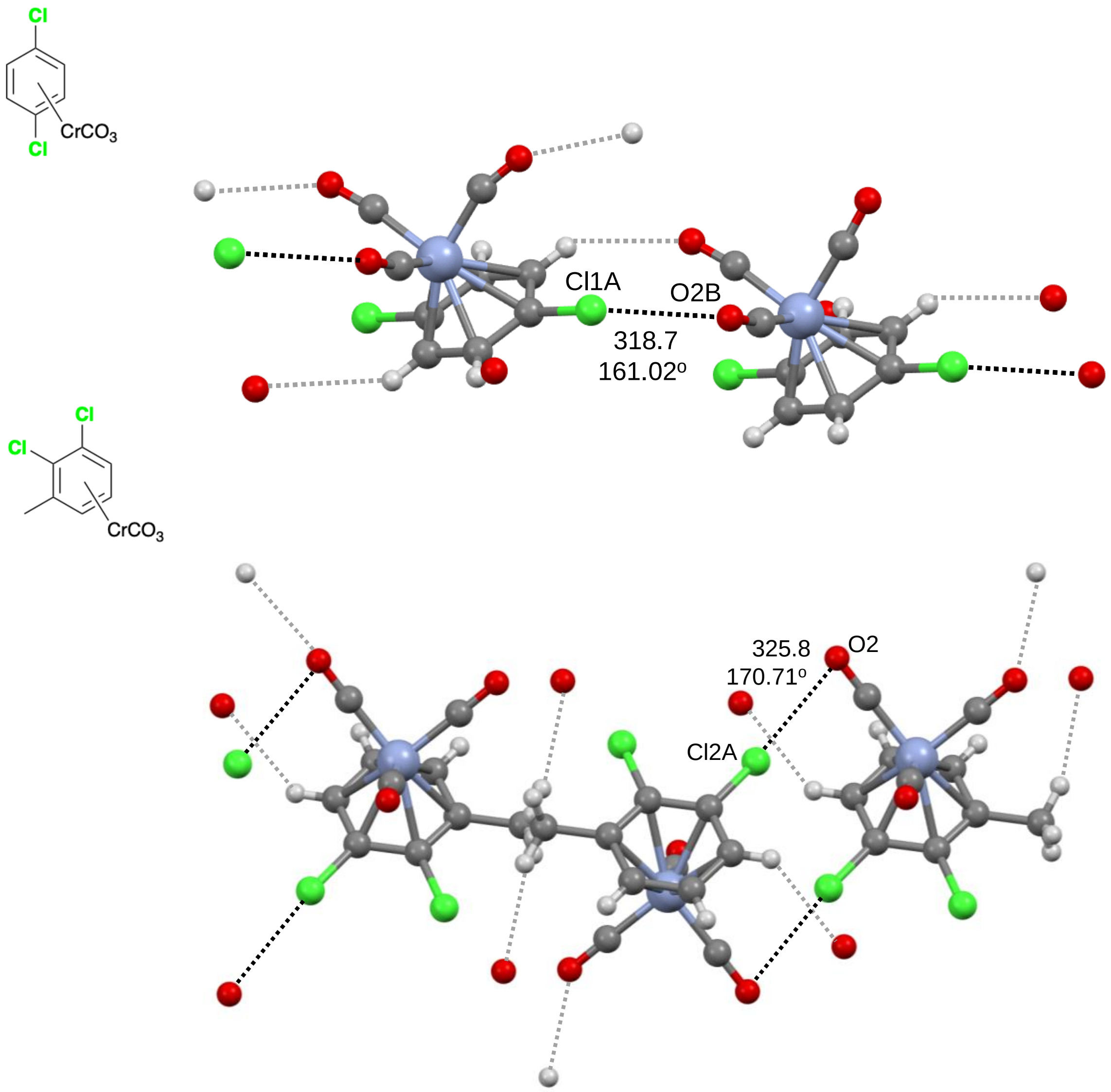

Fig. S5. Partial view (ball and stick representation, Mercury) of the crystal packing of complexes **5** (top) and **6** (bottom). In the complex **6**, only one occupancy of those used for modelling the disorder of one of the crystallographically independent chloroanisole molecules has been reported in the figure for sake of clarity. Cl···O halogen bonds are black dotted lines; H···Cl and H···O hydrogen bonds are grey dotted lines. Cl···O separations (pm) and C–Cl···O angles (°) are reported close to the interactions. Color coding: grey, carbon; whitish, hydrogen; red, oxygen; sky blue, chromium; green, chlorine.

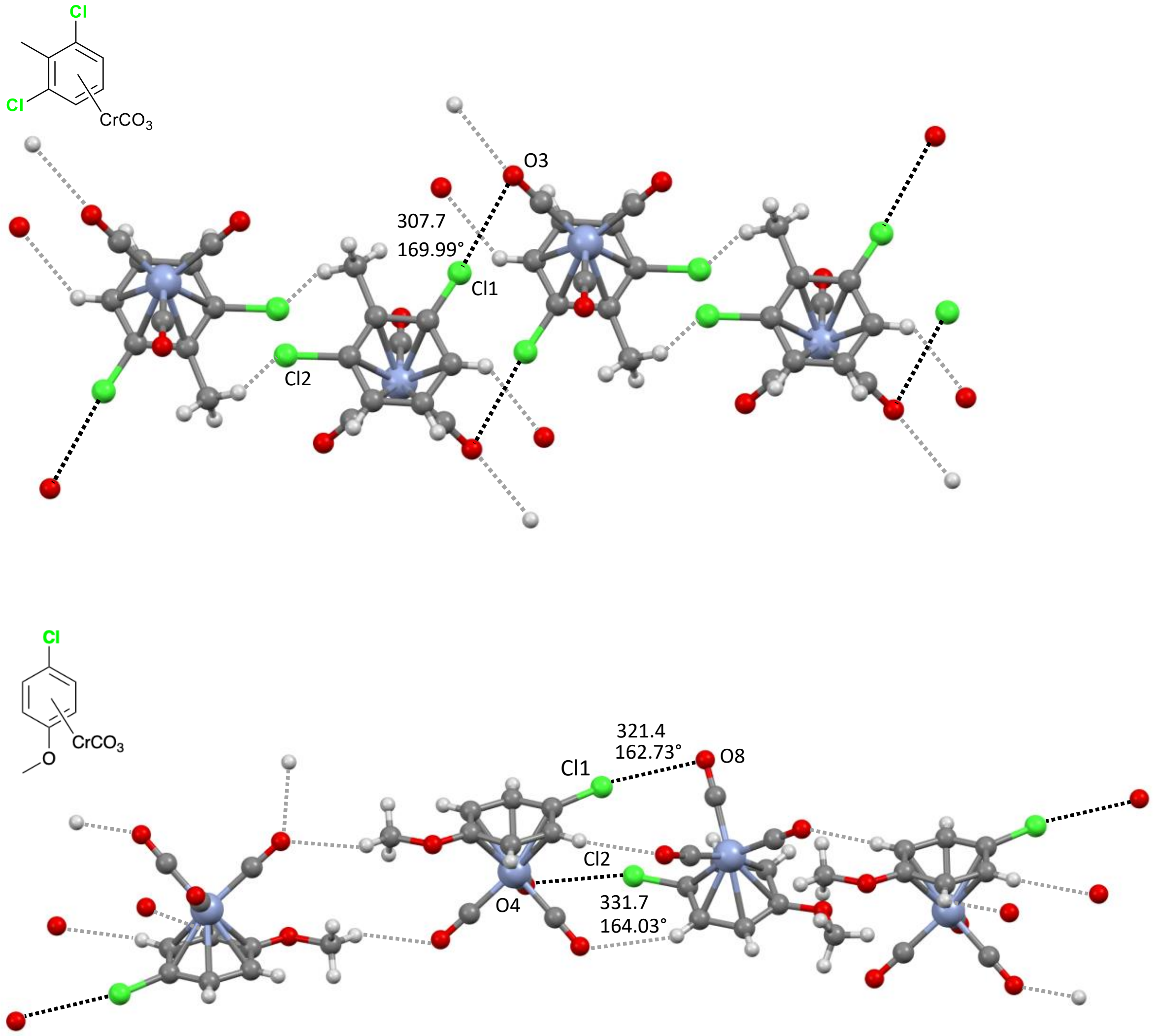

S4. Computational analyses of chloro- and fluoroarene-$Cr(CO)_3$ complexes 1-9 and of complexes 12-15

Fig. S6. Electron density isosurface (ED=0.001 a.u.) mapped by Molecular Electrostatic Potential values for η^6-chloroarene complexes **1–3** (right) and respective uncomplexed chloroarenes (left). Maximal values ($V_{s,max}$) are given. All geometries were optimized in the gas phase with relativistic (spin-orbit) corrections and checked on the absence of imaginary frequencies (for better demonstrate the effect of σ-hole activation).

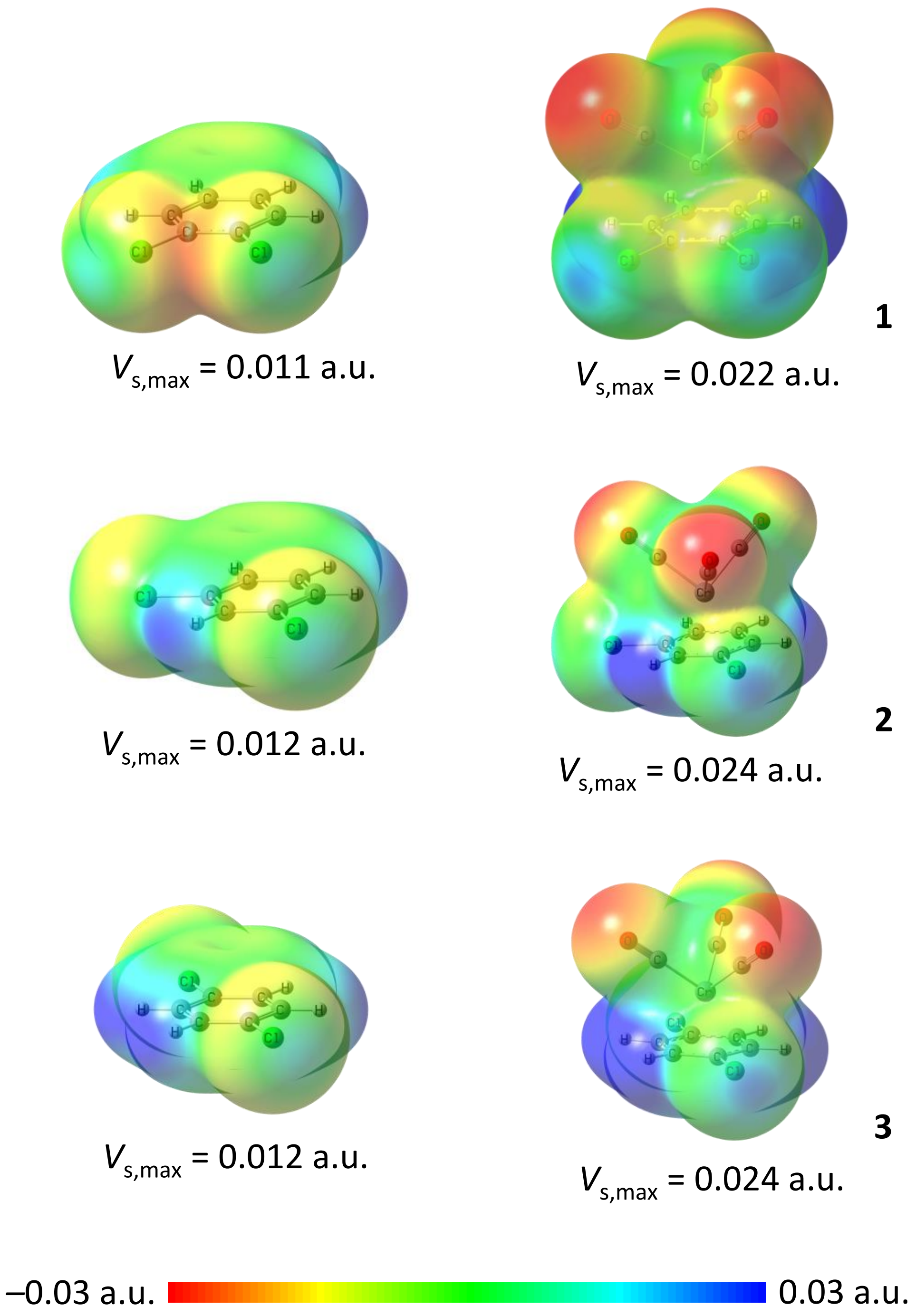

Fig. S7. Electron density isosurface (ED=0.001 a.u.) mapped by Molecular Electrostatic Potential values for η[6]-chloroarene complexes **4–6** (right) and respective uncomplexed chloroarenes (left). Maximal values ($V_{s,max}$) are given. All geometries were optimized in the gas phase with relativistic (spin-orbit) corrections and checked on the absence of imaginary frequencies (for better demonstrate the effect of σ-hole activation).

Das Bild kann zurzeit nicht angezeigt werden.

Fig. S8. IRI (1.0) iso-surface mapped by sign(λ_2)ρ for dimers of η^6-chloroarene-$Cr(CO)_3$ complexes **1**-**6**; adopted geometries are those of the crystals without optimization (in order to establish the features of interaction as in the crystals). The electron density for these fragments was obtained at the same level of theory of Figs. S5-S6. Bond paths are dashed lines; red or orange regions correspond to attraction, blue regions to repulsion.

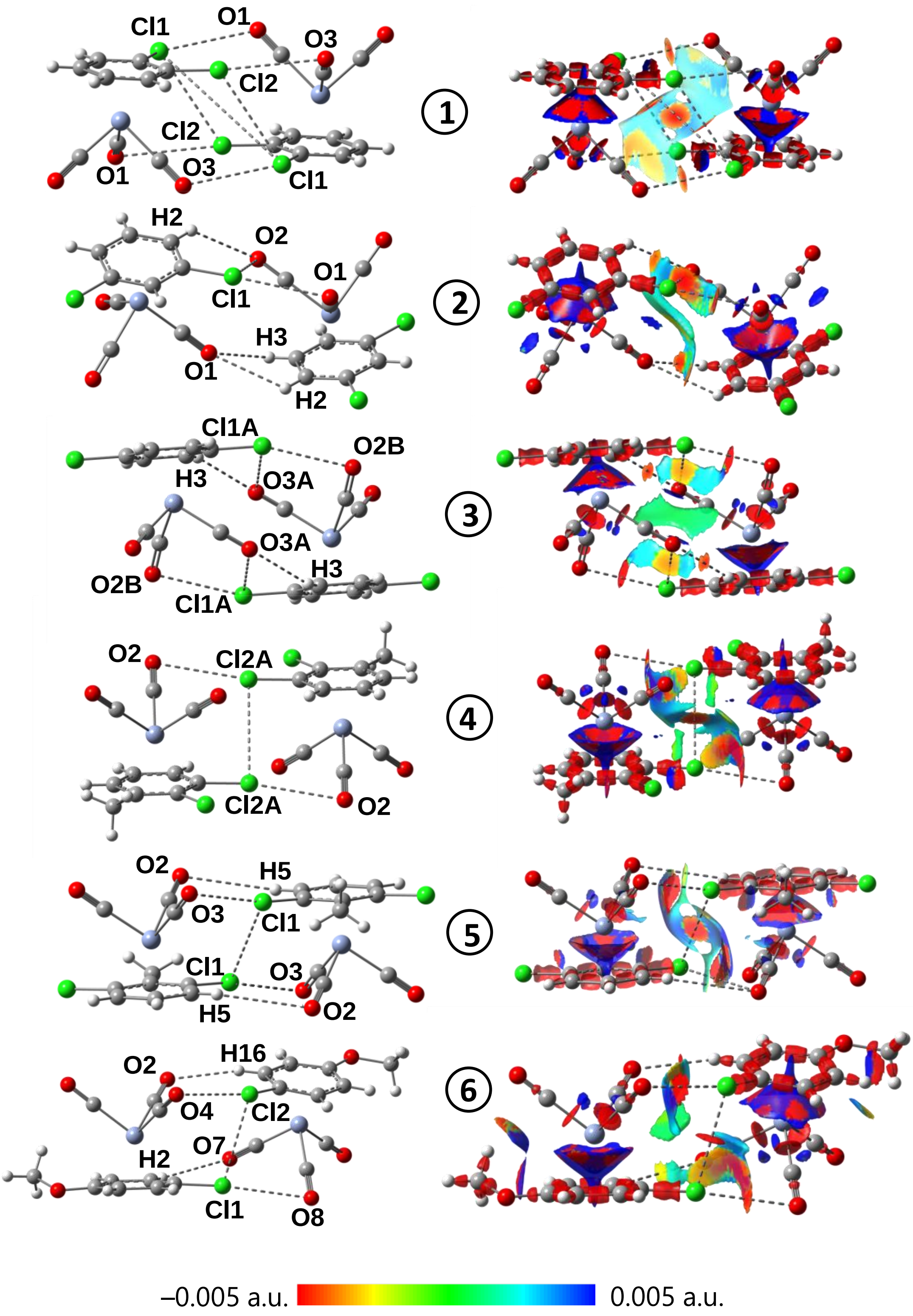

Table S8. QTAIM analysis of optimized dimers of η^6-chloroarene-Cr(CO)$_3$ **1-6** (the atom numbering is that of the .cif file).

	***ρ*, a.u.**	**∇^2*ρ*, a.u.**	***G*, a.u.**	***V*, a.u.**	***H*, a.u.**	***ρ* ellipticity**
**1**						
**Cl1···O1**	0.0035	0.0148	0.0027	–0.0017	0.0010	10.7
**Cl2···O3**	0.0045	0.0193	0.0035	–0.0022	0.0013	0.1
**Cl1···Cl2**	0.0049	0.0170	0.0032	–0.0021	0.0011	0.1
**C1l···Cl1**	0.0047	0.0161	0.0030	–0.0020	0.0010	0.2
**2**						
**Cl1···O1**	0.0052	0.0244	0.0045	–0.0028	0.0016	0.4
**Cl1···O2**	0.0049	0.0197	0.0036	–0.0023	0.0013	0.6
**C-H2···O1**	0.0042	0.0180	0.0034	–0.0023	0.0011	0.1
**C-H2···O2**	0.0046	0.0209	0.0040	–0.0027	0.0013	0.4
**C-H3···O1**	0.0044	0.0208	0.0039	–0.0026	0.0013	0.4
**3**						
**Cl1A···O3A**	0.0036	0.0140	0.0025	–0.0016	0.0010	0.3
**Cl1A···O2B**	0.0064	0.0288	0.0054	–0.0036	0.0018	0.1
**C-H3···O3A**	0.0047	0.0203	0.0039	–0.0026	0.0012	0.0
**4**						
**Cl2A···O2**	0.0059	0.0263	0.0050	–0.0033	0.0016	8.6
**Cl2A···Cl2A**	0.0050	0.0171	0.0032	–0.0022	0.0011	0.1
**5**						
**Cl1···O3**	0.0093	0.0389	0.0077	–0.0058	0.0020	8.7
**C-H5···O2**	0.0018	0.0070	0.0013	–0.0008	0.0005	1.1
**Cl1···Cl1**	0.0052	0.0180	0.0034	–0.0023	0.0011	0.0
**6**						
**Cl1···O8**	0.0062	0.0284	0.0053	–0.0035	0.0018	0.6
**Cl2···O4**	0.0054	0.0230	0.0043	–0.0029	0.0014	5.4
**Cl1···Cl2**	0.0085	0.0304	0.0060	–0.0044	0.0016	0.1
**C-H2···O7**	0.0057	0.0231	0.0044	–0.0031	0.0013	0.0
**C-H16···O2**	0.0024	0.0094	0.0018	–0.0011	0.0006	0.1

Fig. S9. Ball and stick representation (Mercury) of the infinite chain assembled in crystals of complexes **7** (top, Refcode QOBQUU) and **8** (bottom, Refcode QOBPON) via halogen bonds (black dotted lines). Halogen···O separations (pm) and C–halogen···O angles (°) are reported close to the interactions. Color coding: grey, carbon; whitish, hydrogen; red, oxygen; sky blue, chromium; blue, nitrogen; green, chlorine; yellowish green, fluorine.

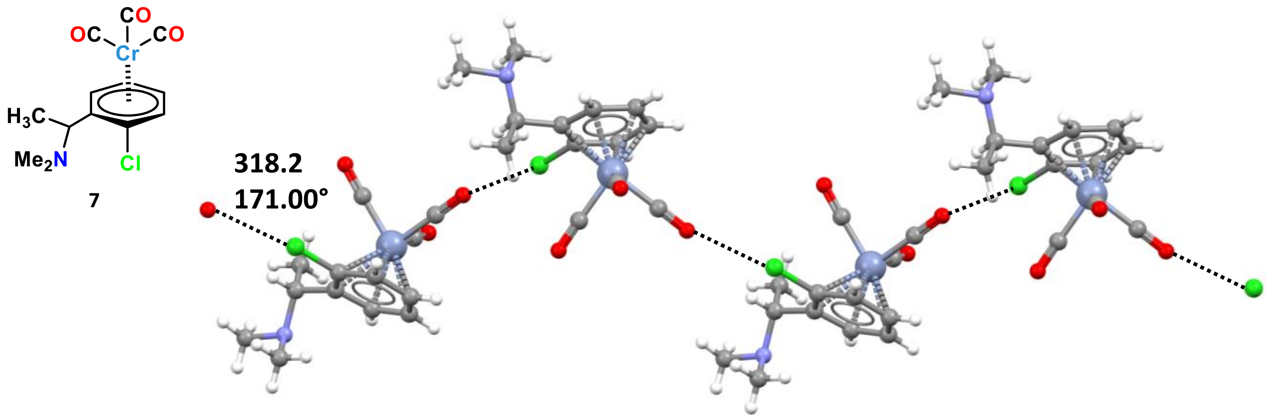

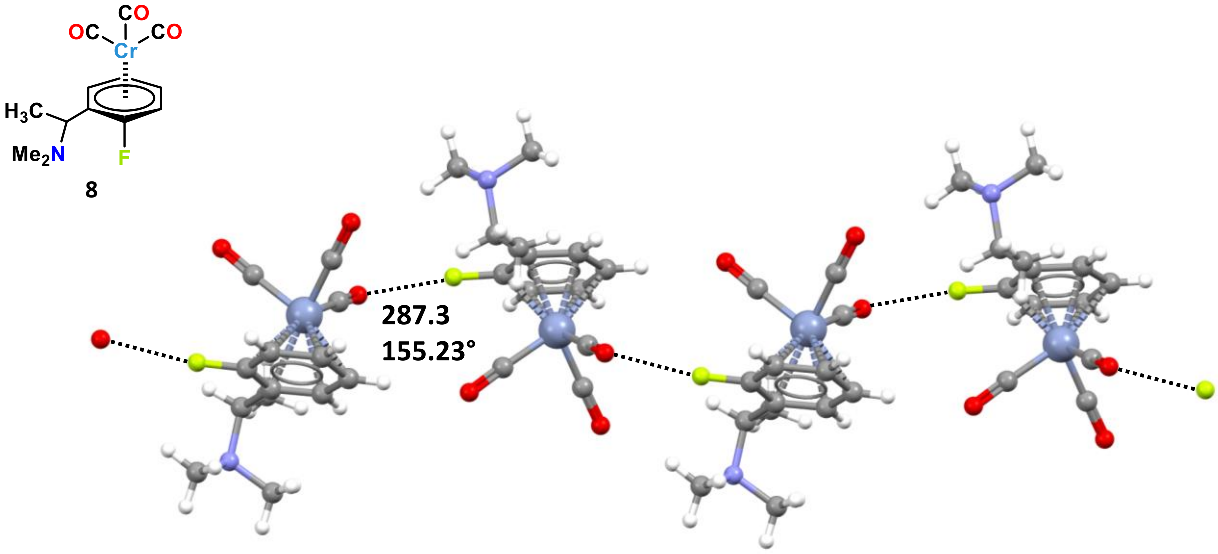

Fig. S10. Ball and stick representation (Mercury) of the infinite chain assembled in complex **9** (Refcode NOMLAD) via halogen bonds (black dotted lines). F···O separation (pm) and C–F···O angle (°) are reported close to the interaction. Color coding: grey, carbon; whitish, hydrogen; red, oxygen; sky blue, chromium; yellowish green, fluorine; orange, phosphorous; pink, boron.

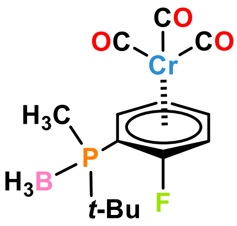

9

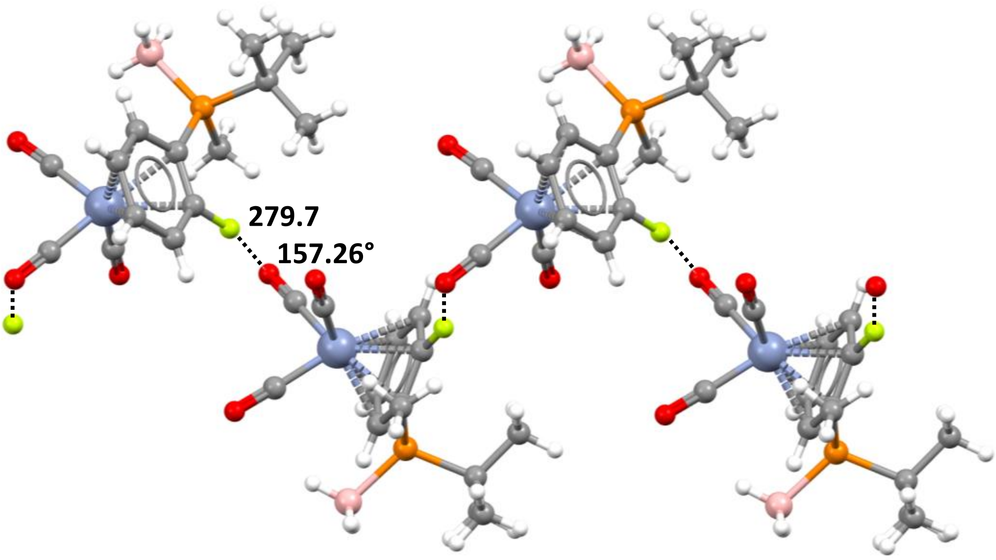

Fig. S11. Geometry of **9** dimer cut from the equilibrated unit cell. Top: IRI isosurface (isovalue 1.0 a.u.) in the proximity of the F···O contact mapped by ED value, ED parameters in CPs of the F···O contact. The values of sign(λ_2)ρ, Laplacian of electron density $\nabla^2\rho$, densities of local kinetic and potential electronic energies densities G and V (in blue) confirm the weak attractive nature of the F···O interaction. Bottom left: Distribution of ELF in C–F···O plane of **9** dimers. Bond paths are white lines, electron density (3, −1) CPs are blue dots, (3, −3) CPs brown dots. Bottom right: Distribution of ED (blue) and ESP (red) along the F···O bond path for **9** dimer. Distance between ED and ESP minimum is given above the arrows.

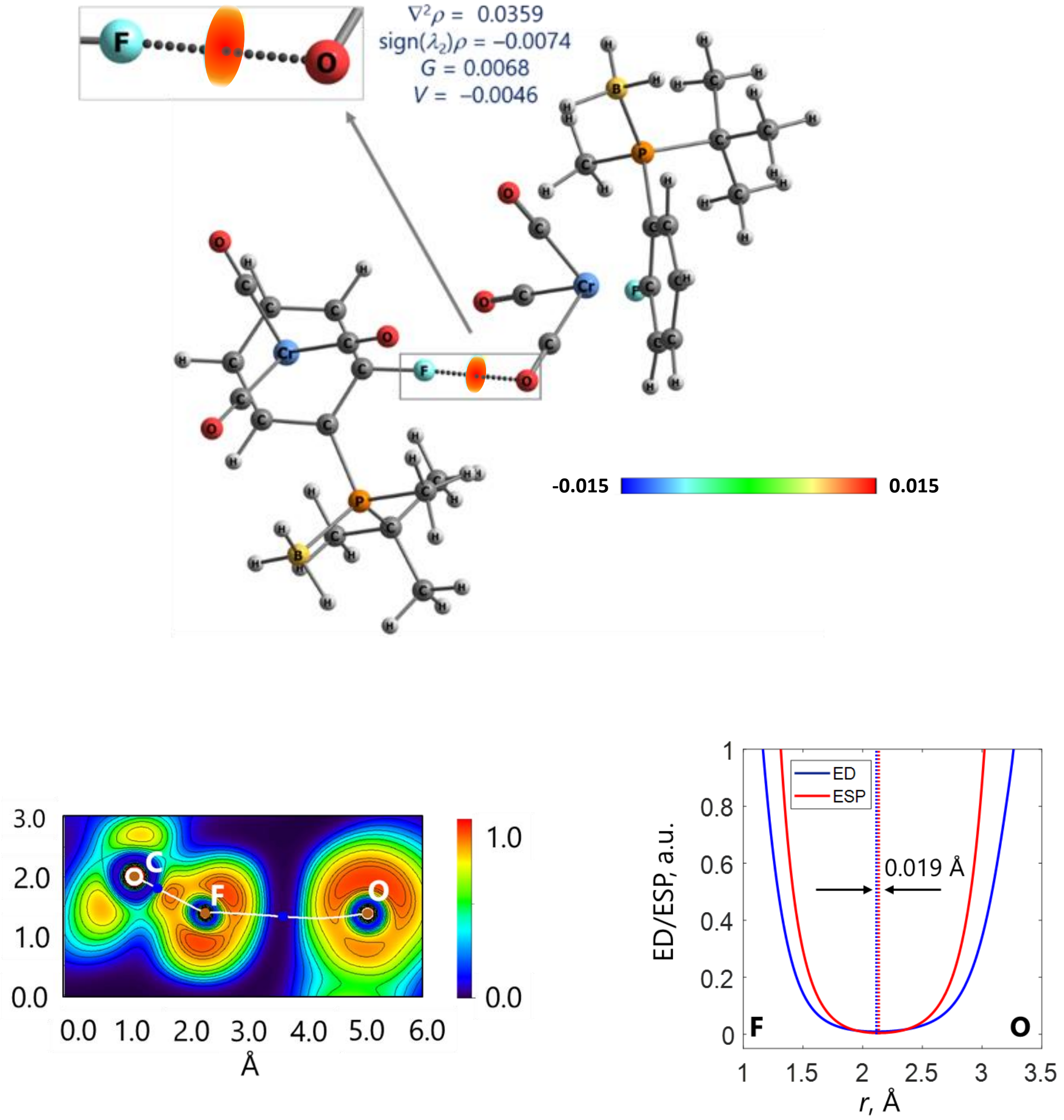

Fig. S12. Molecular structures of 1,2-bis(ethylthio)benzene (left column, top row), its selenium (left column, mid row) and tellurium (left column, bottom row) analogues. Electron density isosurface (ED=0.001 a.u.) mapped by Molecular Electrostatic Potential values for 1,2-bis(ethylthio)benzene (mid column, top row), its selenium (mid column, mid row), tellurium (mid column, bottom row) analogues, and for the η^6-arene complexes **12** (right column, top row), **13** (right column, mid row), and **14** (right column, bottom row). Potential on the elongation of the C_{Ar}–S/Se/Te covalent bond are given. Experimental geometries (from X-ray structure) were used for **12**, for the selenium and tellurium analogs **13** and **14**, the C-–Se and C–Te interatomic distances were further optimized, and the rest of the molecular skeleton was frozen. Calculations were made at the PW6B95-GD3-DKH/jorge-TZP-DKH level of theory.

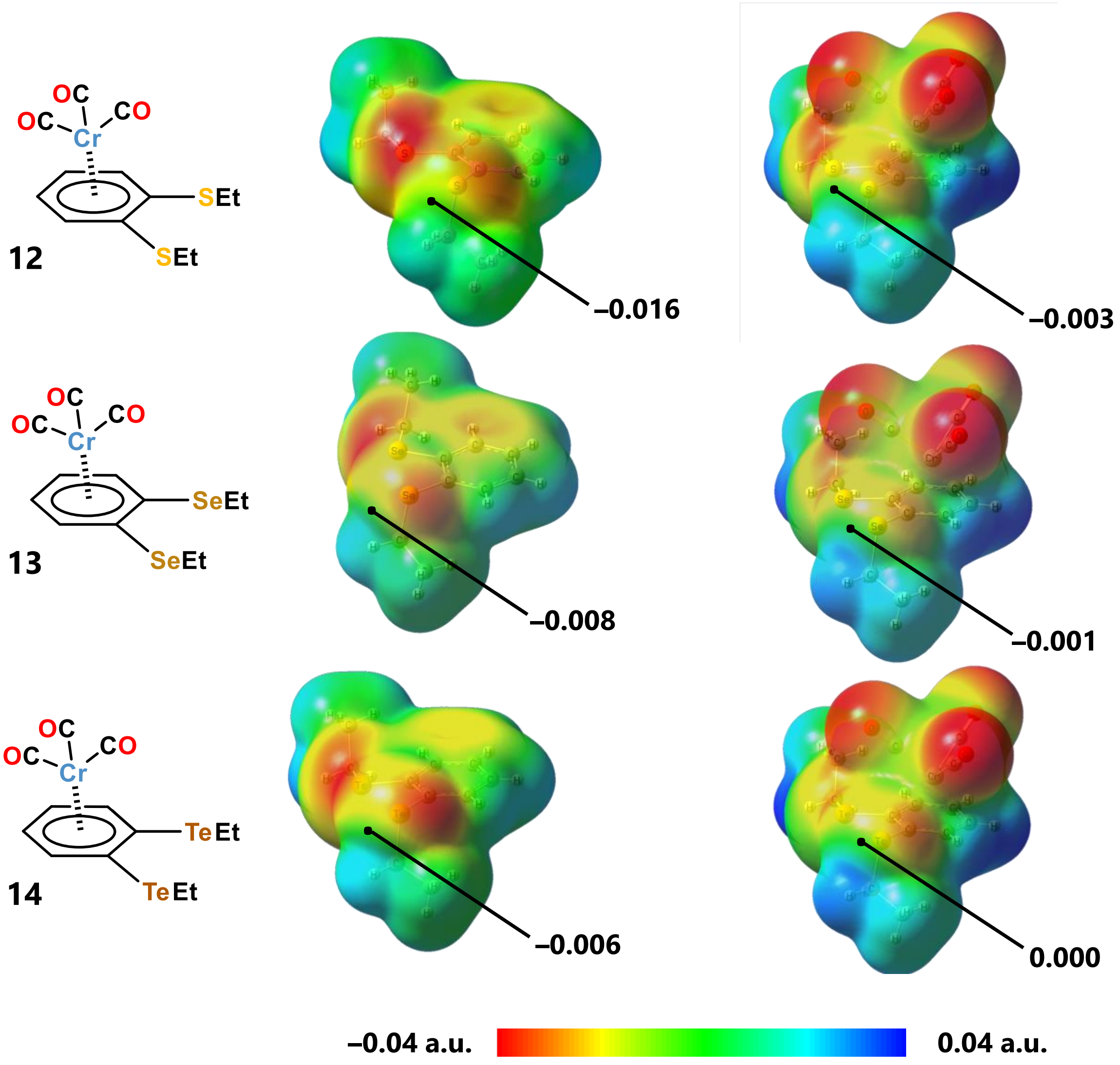

Fig. S13. Ball and stick representation (Mercury) of bis-(η^6-chromium tricarbonyl)triphenyl-bismuthane **15** (Refcode BAYLOC); the pnictogen bond locking the conformation is a violet dotted line; Bi···O separation (pm) and C–Bi···O angle (°) are reported close to the interaction. Color coding: grey, carbon; red, oxygen; sky blue, chromium; violet, bismuth.

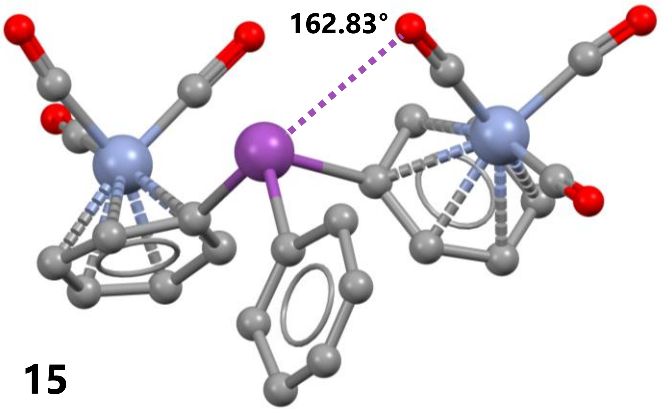

15
